# Efficacy and safety of urate-lowering agents in asymptomatic hyperuricemia: systematic review and network meta-analysis of randomized controlled trials

**DOI:** 10.1186/s12882-022-02850-3

**Published:** 2022-06-23

**Authors:** Tunlanut Sapankaew, Kunlawat Thadanipon, Narisa Ruenroengbun, Kamolpat Chaiyakittisopon, Atiporn Ingsathit, Pawin Numthavaj, Nathorn Chaiyakunapruk, Gareth McKay, John Attia, Ammarin Thakkinstian

**Affiliations:** 1 Department of Clinical Epidemiology and Biostatistics, Faculty of Medicine Ramathibodi Hospital, Mahidol University, Bangkok, Thailand; 2 Faculty of Pharmacy, Silpakorn University, Nakorn Pathom, Thailand; 3 Department of Pharmacotherapy, College of Pharmacy, University of Utah, Salt Lake City, Utah, USA; 4 Centre for Public Health, Queen’s University Belfast, Belfast, UK; 5 Centre for Clinical Epidemiology and Biostatistics, School of Medicine and Public Health, University of Newcastle, Newcastle, NSW Australia

**Keywords:** Systematic review, Network meta-analysis, Treatment, Hyperuricemia, Cardiovascular events, Chronic kidney disease

## Abstract

**Background:**

Asymptomatic hyperuricemia was found to be associated with increased cardiovascular disease risk but the potential benefits of urate-lowering therapy (ULT) remain controversial. We conducted a systematic review and network meta-analysis (NMA) with frequentist model to estimate the efficacy and safety of ULT in asymptomatic hyperuricemia.

**Methods:**

MEDLINE, Embase, and Scopus were searched without language restrictions. Randomized controlled trials (RCT) of adults with asymptomatic hyperuricemia were eligible if they compared any pair of ULTs (i.e., allopurinol, febuxostat, probenecid, benzbromarone, sulfinpyrazone, rasburicase, lesinurad, and topiroxostat) and placebo or no ULT, and had outcomes of interest, including composite renal events, major adverse cardiovascular events, serum urate levels, estimated glomerular filtration rate (eGFR), systolic blood pressure, and adverse events.

**Results:**

NMA with frequentist approach was applied to estimate relative treatment effects, i.e., risk ratio (RR) and mean difference (MD). A total of 23 RCTs were eligible. NMA identified beneficial effects of ULT on composite renal events and eGFR but not for other outcomes. Allopurinol and febuxostat had significantly lower composite renal events than placebo (RR 0.39, 95% confidence interval [CI] 0.23 to 0.66, and RR 0.68, 95% CI 0.46 to 0.99, respectively). Both treatments also resulted in significantly higher eGFR than placebo (MD 3.69 ml/min/1.73 m^2^, 95% CI 1.31 to 6.08, and MD 2.89 ml/min/1.73 m^2^, 95% CI 0.69 to 5.09, respectively). No evidence of inconsistency was identified.

**Conclusions:**

Evidence suggests that allopurinol and febuxostat are the ULTs of choice in reducing composite renal events and improving renal function.

Trial registration.

This study was registered with PROSPERO: CRD42019145908. The date of the first registration was 12^th^ November 2019.

**Supplementary Information:**

The online version contains supplementary material available at 10.1186/s12882-022-02850-3.

## Background

Hyperuricemia is generally defined by serum urate levels (SU) exceeding 6.8 mg/dl [1] and is associated with multiple metabolic comorbidities and premature mortality. Hyperuricemia can span the spectrum from asymptomatic through to various degrees of symptoms such as gout, tophi, and kidney stones [2, 3]. In addition, although hyperuricemia does not always develop into gout, it has been found to be associated with increased risk of cardiovascular disease (CVD) (e.g., hypertension [4, 5], heart failure, coronary artery disease [6], atrial fibrillation [7], and acute stroke [8]), as well as acute kidney injury [9], chronic kidney disease (CKD) [10, 11], and greater decline in renal function [12].

The prevalence of asymptomatic hyperuricemia is relatively common, ranging from 10.6% to 25.8% in the general population [13–16]. However, treatment recommendations vary from no treatment [1, 17, 18] through to use of urate-lowering therapy (ULT) to minimize comorbidities (e.g., Japanese guidelines [19]). This may partly be due to the differences in supporting primary research evidence, such as clinical trials and observational studies, and variety of expert opinions in each organization or country. This allowed clinicians to use their own judgement to prescribe ULT in asymptomatic hyperuricemic patients.

To our knowledge, 5 pairwise meta-analyses [20–24] and a single network meta-analysis (NMA) [25] have previously focused on the treatment of mixed asymptomatic and symptomatic hyperuricemia but none have considered only asymptomatic hyperuricemia. Given the publication of several recent trials of ULT in asymptomatic hyperuricemia were inconsistent. Some studies showed the reno-protective benefit of ULTs in asymptomatic patients but some studies did not [26–29]. The need for a systematic review and NMA to evaluate the potential benefits of ULT against several outcome measures was apparent.

## Methods

This study was performed in accordance with the Preferred Reports of Systematic Review and Meta-Analysis (PRISMA) 2020 Statement [30, 31] and was registered in PROSPERO (CRD42019145908).

### Data Sources and Searches

Studies were identified through MEDLINE via PubMed, Scopus, and Embase from inception to June 2019, using the search terms described in Additional file: Table S1. All studies identified were independently selected by 2 of 3 reviewers (TS, NR, and KC).

Titles and abstracts were screened first, and full texts were retrieved and reviewed if decision of inclusion could not be made. Any disagreement was discussed with a third party (AI). Randomized controlled trials (RCT) were included where they met the following criteria: enrolled participants aged 18 years or older with hyperuricemia, compared ULTs (i.e., allopurinol, febuxostat, probenecid, benzbromarone, rasburicase, sulfinpyrazone, lesinurad, and topiroxostat) and placebo or no ULT, and reported any of the outcomes of interest. RCTs were excluded if they included patients with symptomatic (e.g., with gouty arthritis, stones, or tophi) or secondary hyperuricemia (e.g., tumor lysis syndrome and drug-induced hyperuricemia), or had insufficient data for pooling following 3 attempts to contact authors via email. Missing data was not imputed.

The primary outcomes of interest included composite renal events and major adverse cardiac events (MACE) defined in accordance with the original RCTs. Composite renal events included deterioration of renal function, end-stage renal disease, and initiation of renal replacement therapy, but excluded the development of isolated albuminuria. MACE was defined as cardiovascular death, myocardial infarction, stroke, and hospitalization due to heart failure.

The secondary surrogate outcomes included SU, estimated glomerular filtration rate (eGFR), systolic blood pressure (SBP), and composite adverse events (AEs), including elevated liver enzymes, gouty attack, rash, and gastrointestinal symptoms.

### Data Extraction and Quality Assessment

Data extraction was performed by the reviewers that identified the studies. The extracted data comprised: dosage, duration, comorbidity, baseline laboratory values, number of participants, and outcome types. Outcome data (i.e., event numbers, mean, and standard deviation [SD]) were extracted by ULT groups based on an intention-to-treat approach. If individual outcome data were reported instead of composite outcome data, the maximum number of individual outcomes were extracted and used for quantitative analysis. Any disagreement was discussed and resolved by a third party (AI).

The quality of studies was assessed independently by the same reviewers using the revised Cochrane risk of bias tool (RoB 2) [32]. An overall risk of bias was finally rated as low risk, some concerns, and high risk. Disagreements were assessed by kappa statistic and resolved by consensus with a third party (AI).

### Data Synthesis and Analysis

In pairwise meta-analysis, relative treatment effects (i.e., risk ratio [RR] and mean difference [MD] for dichotomous and continuous outcomes, respectively) were estimated and pooled across the studies using a random-effects model if heterogeneity was present, otherwise a fixed-effect model was used. The I^2^ statistic and Q test were applied; heterogeneity was present if the I^2^ value ≥ 25% or *P*-value from the Q test < 0.1. The source of heterogeneity was explored by fitting each covariate in a meta-regression model. If the I^2^ value was decreased by 50% or more, a subgroup analysis by that covariate was undertaken.

Two-stage frequentist NMA with consistency model [33, 34] was applied to estimate the relative treatment effects of all ULTs. The ln(RR) or MD and its variance–covariance were estimated for each study using a placebo measure as the common comparator. These were pooled across studies using a multivariate meta-analysis with consistency model and multiple treatment contrasts were estimated. The surface under the cumulative ranking curve (SUCRA) was applied to rank treatments by the maximum probability on the basis of efficacy and safety. The consistency assumption was assessed using design-by-treatment interaction model [35], and comparison-adjusted funnel plots were used to assess publication bias. The credibility of results from a network meta-analysis was evaluated using novel methodological framework Confidence in Network Meta-Analysis (CINeMA) [36].

For SU, eGFR and SBP, the NMA analyzed allopurinol and febuxostat categorized by dosage in order to prove the effectiveness of dose–response relationship and help practitioners choose the proper ULT upon socioeconomic level of each country. Low-dose allopurinol was defined as < 300 mg/day, high-dose allopurinol as ≥ 300 mg/day, low-dose febuxostat as < 40 mg/day, and high-dose febuxostat as ≥ 40 mg/day.

All analyses were performed using Stata version 16.0 (StataCorp). Statistical significance was considered if a 2-sided *P*-value was < 0.05, except where indicated.

## Results

### Overview of Trials

**Fig. 1:**
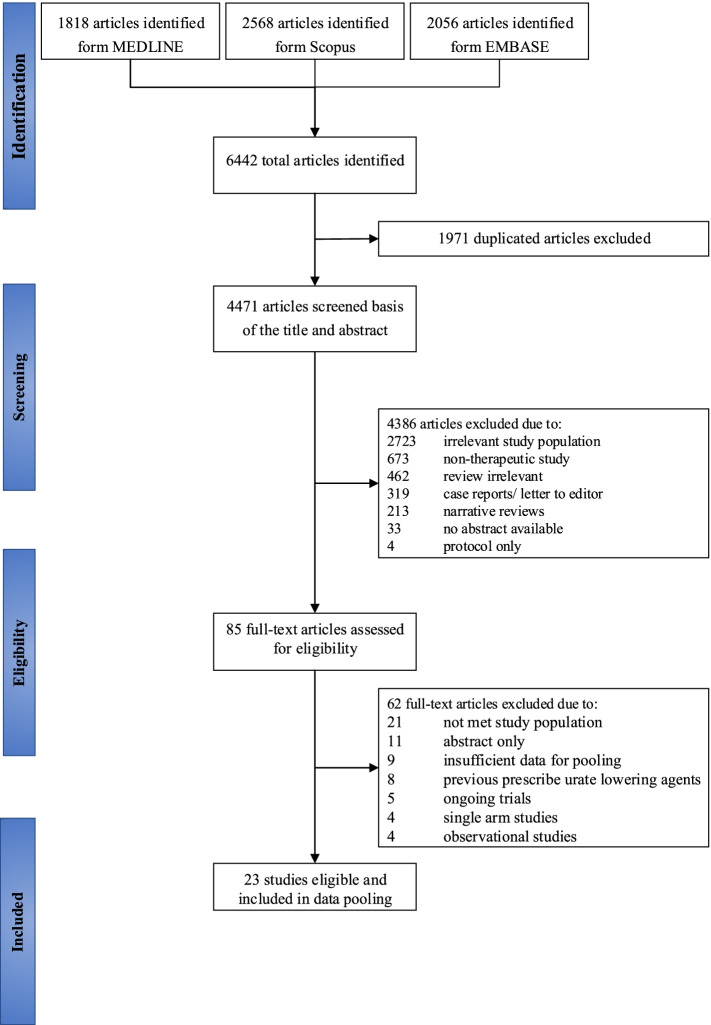
Flow chart of study selection

A total of 23 RCTs (3209 participants) were eligible from 6442 studies (Fig. 1). Of the 23 eligible studies, 19 and 4 were parallel and cross-over RCTs, respectively. Mean age and body mass index were 65.9 years and 26.6 kg/m^2^, and 65.3% of participants were male. The follow-up time ranged from 5 days to 7 years with a median of 5.8 months. Proportion of people with hypertension, diabetes mellitus, CKD, and CVD were 75.4%, 42.4%, 83.4%, and 33.8%, respectively (Table 1, and Additional file: Table S2).

**Table 1:** Characteristics of included studies

**Author**	**Country**	**Year**	**Design**	**Duration** (month)	*n*	**Drugs** (mg)	**Age** (mean)	**M** (%)	**HT** (%)	**DM** (%)	**CKD** (%)	**CVD** (%)
Doehner[37]	England	2002	2-Arm cross-over	0.5	16	Allopurinol (300)—placebo/no ULT	68.5	100	N/A	N/A	N/A	100
Siu[38]	China	2006	2-Arm parallel	12.0	51	Allopurinol (200)—placebo/no ULT	48.3	42.4	78.4	25.5	100.0	N/A
Ogino[56]	Japan	2010	2-Arm cross-over	1.9	14	Benzbromarone (100)—placebo/no ULT	60.0	71.4	21.4	N/A	N/A	100
Kanbay[39]	Turkey	2011	3-Arm parallel	4.0	67	Allopurinol (300)—placebo/no ULT	52.9	51	N/A	N/A	N/A	N/A
Jalalzadeh[57]	Iran	2012	2-Arm cross-over	3.0	55	Allopurinol (100)—placebo/no ULT	55.9	62.3	100.0	N/A	100.0	N/A
Ejaz[26]	USA	2013	2-Arm parallel	0.2	26	Rasburicase (7.5)—placebo/no ULT	62.4	69.2	100	30.8	100.0	34.7
Sezai[58]	Japan	2013	2-Arm parallel	6.0	141	Febuxostat (60)—allopurinol (300)	66.9	82.1	80.7	36.4	77.9	100
Taheraghdam[59]	Iran	2014	2-Arm parallel	3.0	65	Allopurinol (200)—placebo/no ULT	69.1	36.0	36.0	63.5	N/A	41.5
Goicoechea[41]	Spain	2015	2-Arm parallel	84.0	113	Allopurinol (100)—placebo/no ULT	71.8	N/A	N/A	N/A	100.0	33.1
Liu[60]	China	2015	2-Arm parallel	36.0	176	Allopurinol (100)—placebo/no ULT	50.5	48.3	N/A	N/A	N/A	N/A
Sircar[61]	India	2015	2-Arm parallel	6.0	108	Febuxostat (40)—placebo/no ULT	57.3	70.5	98	37.5	100.0	37.5
Takir[62]	Turkey	2015	3-Arm parallel	3.0	73	Allopurinol (300)—placebo/no ULT	51.1	49.2	54.7	N/A	N/A	N/A
Nakagomi[63]	Japan	2015	2-Arm parallel	12.0	61	Febuxostat (40)—allopurinol (300)	70.6	69.5	93.7	34.4	N/A	N/A
Tani[64]	Japan	2015	2-Arm parallel	6.0	60	Febuxostat (10)—placebo/no ULT	68.0	93.5	100.0	31.5	43.5	31.5
Tsuruta[65]	Japan	2015	2-Arm parallel	0.9	54	Febuxostat (10)—placebo/no ULT	68.3	64.2	79.3	41.4	100.0	58.5
Beddhu[66]	USA	2016	2-Arm parallel	5.6	80	Febuxostat (80)—placebo/no ULT	63.5	35	77.5	100.0	100.0	36.3
Kojima[67]	Japan	2016	2-Arm cross-over	0.9	14	Benzbromarone (400)—allopurinol (25)	70.0	71.0	100	N/A	N/A	N/A
Golmohammadi[68]	Iran	2017	2-Arm parallel	12.0	196	Allopurinol (100)—placebo/no ULT	N/A	54.6	60.7	40.3	100	N/A
McMullan[69]	USA	2017	3-Arm parallel	1.9	149	Probenecid (500)—allopurinol (300)—placebo/no ULT	40.4	49.7	N/A	N/A	N/A	N/A
Jalal[70]	USA	2017	2-Arm parallel	2.8	80	Allopurinol (300)—placebo/no ULT	57.4	80	N/A	61.0	100	45.1
Kimura[27]	Japan	2018	2-Arm parallel	25.2	441	Febuxostat (40)—placebo/no ULT	65.4	77.3	34.9	48.0	100	21.8
Mukri[29]	Malaysia	2018	2-Arm parallel	6.0	100	Febuxostat (40)—placebo/no ULT	65.5	53.5	46.5	N/A	100	30
Kojima[28]	Japan	2019	2-Arm parallel	36.0	1070	Febuxostat (40)—allopurinol	75.7	69.4	94.1	37.0	66.1	25.7

*CKD* Chronic kidney disease, *CVD* Cardiovascular disease, *DM* Diabetes mellitus, *HT* Hypertension, *M* Male, *n* Number of participants, *N/A* Not available, *ULT* Urate-lowering therapy, *USA* United States of America

Allopurinol, febuxostat, probenecid, benzbromarone, and rasburicase were studied in 15, 9, 1, 2, and 1 RCTs, respectively. Outcomes of interest included composite renal events (*n* = 8), MACE (*n* = 7), SU (*n* = 21), eGFR (*n* = 11), SBP (*n* = 13), and AEs (*n* = 11).

### Study Quality

Our assessments suggested that 82.6%, 69.5%, 86.9%, 39.1%, and 60.8% of studies demonstrated a low risk of bias for randomization, deviations from intended interventions, missing outcome data, measurement of the outcome, and selection of the reported results, respectively (Additional file: Table S3). As a result, 21.7% and 69.5% of studies expressed a low risk of overall bias and some concerns, respectively. The agreement between 2 of the 3 reviewers was 90.6% (kappa = 0.82).

### Pairwise Meta-analysis

Allopurinol and febuxostat both reduced the number of renal events but this failed to reach significance relative to the placebo/no ULT (RR 0.42, 95% CI 0.17 to 1.02, and RR 0.69, 95% CI 0.46 to 1.02, respectively) (Additional file: Figure S1). Both treatments also resulted in a reduction of MACE compared to placebo/no ULT (RR 0.70, 95% CI 0.41 to 1.20, and RR 0.87, 95% CI 0.24 to 3.12, respectively). The pooled RR of febuxostat relative to allopurinol was 0.77 (95% CI 0.46 to 1.30) (Additional file: Figure S2).

Comparison of surrogate outcomes indicated significantly reduced SU versus placebo/no ULT for allopurinol (*n *= 9) and febuxostat (*n* = 6) (MD -2.04 mg/dl, 95% CI -2.61 to -1.47, and MD -3.02 mg/dl, 95% CI -3.70 to -2.34, respectively) (Additional file: Figure S3). Febuxostat also demonstrated lower SU than allopurinol (*n* = 3) (MD -1.10 mg/dl, 95% CI -2.45 to 0.25), but this was not significant. In addition, allopurinol (*n* = 4) and febuxostat (*n* = 4) both showed increased eGFR measures (MD 5.30 ml/min/1.73 m^2^, 95% CI 2.64 to 7.99, and MD 1.52 ml/min/1.73 m^2^, 95% CI -0.45 to 3.49, respectively) relative to placebo/no ULT, but the latter was not significant (Additional file: Figure S4). Furthermore, allopurinol (*n* = 7) and febuxostat (*n* = 4) showed lower SBP relative to placebo/no ULT with (MD -4.47 mmHg, 95% CI -9.37 to 0.43, and MD -0.97 mmHg, 95% CI -3.55 to 1.61, respectively), but these also failed to reach significance (Additional file: Figure S5). Both medications showed greater risk of AEs than placebo/no ULT but these were not significant (RR 1.48, 95% CI 0.86 to 2.54, and RR 2.75, 95% CI 0.27 to 28.45, respectively) (Additional file: Figure S6).

Heterogeneity I^2^ values ranged from 0 to high for dichotomous outcomes, i.e., 0% to 38.2%, 0% to 28.2%, and 25.7% to 71.4% for composite renal events, MACE, and AEs, respectively. The I^2^ value was low to very high for continuous outcomes, i.e., 91.1% to 99.3 for SU, 0% to 19.5% for eGFR, and 0% to 83.1% for SBP (Additional file: Table S4).

### Additional analyses

Meta-regression identified that the percentage of males reduced the degree of heterogeneity in MACE and AEs as the percentage of patients with hypertension did in SU (Additional file: Table S5). It was only possible to perform subgroup analysis for SU, with febuxostat showing greater effect in the hypertension ≤ 50% subgroup (*n* = 2) compared to hypertension > 50% (*n* = 4) (MD -3.77 mg/dl, 95% CI -3.99 to -3.56, and MD -2.68 mg/dl, 95% CI -3.27 to -2.08, respectively) (Additional file: Figure S7). Sensitivity analyses excluding study contained all hypertensive patients, febuxostat group had lower SU of -2.92 mg/dl, 95% CI -3.35 to -2.49 with I^2^ of 32.80% (Additional file: Figure S8).

### Network meta-analysis

#### Primary Outcomes

Eight (1991 participants) and 7 (1916 participants) RCTs were included in the NMAs of composite renal events and MACE, respectively. Three ULTs (i.e., allopurinol, febuxostat, and rasburicase) and placebo/no ULT were included in the NMA of composite renal events (Additional file: Figure S9). Probenecid was excluded as it was contraindicated in patients with deteriorating renal function. Allopurinol and febuxostat had significantly lower composite renal events relative to placebo/no ULT (RR 0.39, 95% CI 0.23 to 0.66, and RR 0.68, 95% CI 0.46 to 0.99, respectively), whereas rasburicase was associated with increased risk (RR 1.14, 95% CI 0.59 to 2.22) although this was not significant, see Table 2. Among active ULTs, rasburicase and febuxostat showed a 2.92 (95% CI 1.25 to 6.78) and 1.72 (95% CI 0.94 to 3.17) times greater risk than allopurinol, although the latter was not significant.

**Table 2:** Mixed relative treatment effects of major adverse cardiovascular event and composite renal events among urate lowering agents

MACE; RR (95% CI)			
**Rasburicase**	-	-	-
1.69 (0.79, 3.63)	**Febuxostat**	0.82 (0.52, 1.32)	0.62 (0.35, 1.11)
2.92 (1.25, 6.78)	1.72 (0.94, 3.17)	**Allopurinol**	0.75 (0.47, 1.21)
1.14 (0.59, 2.22)	**0.68** **(0.46, 0.99)**	**0.39** **(0.23, 0.66)**	**Placebo/no ULT**
Composite renal events; RR (95% CI)			

*CI* Confidence interval, *MACE* Major adverse cardiovascular events, *RR* Risk ratio, *ULT*, Urate-lowering therapy agents

Comparisons are read from left to right for both MACE and composite renal events. For example; allopurinol had lower composite renal events with RR (95% CI) of 0.39 (0.23, 0.66) compared with placebo/no ULT, and lower MACE with RR (95% CI) of 0.75 (0.47, 1.21) compared with placebo/no ULT

Bold font indicates statistical significance

Treatment with allopurinol and febuxostat were less likely to lead to MACE compared to placebo/no ULT (RR 0.75, 95% CI 0.47 to 1.21, and RR 0.62, 95% CI 0.35 to 1.11, respectively), although these were not significant, see Table 2. Likewise, febuxostat showed a non-significant reduction in risk of MACE relative to allopurinol (RR 0.82, 95% CI 0.52 to 1.32). There was no evidence of inconsistency for MACE (χ^2^ = 0.26, *P*-value = 0.60) and composite renal events (χ^2^ = 0.07, *P*-value = 0.79).

### Secondary outcomes

Data pooling from 11 (2532 participants), 23 (3063 participants), 13 (1555 participants), and 13 (2493 participants) comparison arms were used in NMAs of eGFR, SU, SBP, and AEs, respectively (Additional file: Figure S10). Allopurinol and febuxostat had significantly higher eGFR than placebo/no ULT (MD 3.69 ml/min/1.73 m^2^, 95% CI 1.31 to 6.08, and MD 2.89 ml/min/1.73 m^2^, 95% CI 0.69 to 5.09, respectively) (Table 3 and Additional file: Table S6).

**Table 3:** Mixed relative treatment effects of composite adverse events and estimated glomerular filtration rate among urate lowering agents

eGFR; MD (95% CI)			
**Probenecid**	-	-	-
0.52 (0.08, 3.52)	**Febuxostat**	-0.80 (-2.66, 1.05)	**2.89** **(0.69, 5.09)**
0.42 (0.10, 1.73)	0.80 (0.23, 2.82)	**Allopurinol**	**3.69** **(1.31, 6.08)**
0.68 (0.16, 2.84)	1.30 (0.28, 5.99)	1.63 (0.70, 3.79)	**Placebo/no ULT**
Adverse events; RR (95% CI)			

*CI* Confidence interval, *eGFR* Estimated glomerular filtration rates, *MD* Mean difference, *RR* Risk ratio, *ULT* Urate-lowering therapy agents

Comparisons are read from left to right for both eGFR and adverse events. For example; allopurinol had higher adverse events with RR (95% CI) of 1.63 (0.70, 3.79) compared with placebo/no ULT, and higher eGFR with MD (95% CI) of 3.69 (1.31, 6.08) compared with placebo/no ULT

Bold font indicates statistical significance

SU levels were compared between 7 interventions (Additional file: Figure S10). All ULTs resulted in significantly lower SU than placebo/no ULT (MD -4.30 mg/dl, 95% CI, -6.32 to -2.27 for rasburicase, MD -3.29 mg/dl, 95% CI -4.07 to -2.51 for high-dose febuxostat, MD -2.49 mg/dl, 95% CI -3.66 to -1.31 for uricosuric agents [i.e., probenecid and benzbromarone], MD -2.45 mg/dl, 95% CI -3.85 to -1.04 for low-dose febuxostat, MD -2.45 mg/dl, 95% CI -3.21 to -1.70 for high-dose allopurinol, and MD -1.63 mg/dl, 95% CI -2.51 to -0.74 for low-dose allopurinol) (Additional file: Table S7). Among active ULTs, rasburicase and high-dose febuxostat resulted in significantly reduced SU compared to low-dose allopurinol (MD -2.67 mg/dl, 95% CI -4.88 to -0.46, and MD -1.66 mg/dl, 95% CI -2.73 to -0.60, respectively). However, high dose and low dose of the same treatments did not significantly differ (MD -0.83 mg/dl, 95% CI -1.96 to 0.30 for allopurinol, and MD -0.84 mg/dl, 95% CI -2.45 to 0.76 for febuxostat).

Four interventions were included in the NMA of SBP, i.e., allopurinol, febuxostat, probenecid, and placebo/no ULT (Additional file: Figure S10). Allopurinol and febuxostat lowered SBP by approximately 3 and 2 mmHg, respectively, whereas probenecid showed little MD compared to placebo/no ULT, although none of the comparisons were significant (Additional file: Table S8 and Table S9).

Four interventions were included for comparisons of AEs, i.e., allopurinol, febuxostat, probenecid, and placebo/no ULT (Additional file: Figure S10). Allopurinol and febuxostat showed a higher risk of AEs compared to placebo/no ULT (RR 1.63, 95% CI 0.70 to 3.79, and RR 1.30, 95% CI 0.28 to 5.99, respectively) while probenecid demonstrated a lower risk (RR 0.68, 95% CI 0.16 to 2.84); however, none of these associations were significant (Table 3). In addition, probenecid had a lower risk compared to allopurinol (RR 0.42, 95% CI 0.10 to 1.73) but again, this was not significant.

Consistency assumption checks for all NMAs indicated no evidence of inconsistency. Comparison-adjusted funnel plots also showed no evidence of publication bias for most NMAs (Additional file: Figure S11). The ranking of treatments by efficacy and AEs through SUCRAs were displayed in the scatter plot on the x and y axes, respectively (Additional file: Table S12 and Figure S12). Allopurinol and febuxostat were the most efficacious for composite renal events, MACE, preserving eGFR, but showed higher risk of AEs compared to probenecid and placebo/no ULT. The confidence in the results of NMA were assessed using CINeMA approach, the confidence rating varied from moderately to very low. (Additional file: Table S13).

## Discussion

A systematic review and NMA was performed and included 23 RCTs to assess the efficacy and AEs associated with the use of ULTs for the treatment of asymptomatic hyperuricemia. The findings highlight the beneficial effects of allopurinol and febuxostat in lowering both the frequency of composite renal events and the rate of decline in renal function (eGFR) compared to placebo/no ULT. Lower SU however, were not associated with a significant reduction in cardiovascular events (MACE) or lower SBP. Furthermore, there were no significant differences in AEs associated with ULTs compared to placebo/no ULT groups.

Our findings also showed that rasburicase might be the most efficacious ULT, followed by high-dose febuxostat, uricosuric agents, and high-dose allopurinol, for SU reduction. The treatment effects observed between high- and low-dose ULTs were not significantly different and a sensitivity analysis that excluded rasburicase still showed significantly lower composite renal events from allopurinol and febuxostat compared to placebo/no ULT controls (Additional file: Table S10 and Table S11). Our findings suggest that allopurinol and febuxostat can reduce further composite renal events and slow progression of CKD, supporting previous studies for allopurinol [37–40] and febuxostat [22, 27, 28, 41, 42].

Despite the significant associations between ULTs and renal outcomes, we found no beneficial use of ULTs with regards to MACE and SBP outcomes, similar to reports that xanthine oxidase inhibitors (XOI) did not reduce cardiovascular events [43]. In contrast, Singh et al. [44] reported reduced risk of incident myocardial infarction with allopurinol among the elderly, possibly as a result of its anti-ischemic mode of action [45] and reduced CD36-mediated TLR4/6-IRAK4/1 signaling[46]. Given its previous association with increased cardiac risk [47], reduction of SU should be considered in parallel with other notable risk factors such as SBP, blood sugar, and dietary behavior to limit the number of MACE. Allopurinol has been reported to inhibit xanthine oxidase in the early stages of purine metabolism, leading to reduced oxidative stress, improved endothelial function, and reduced glomerular hypertension [48, 49].

Febuxostat is a viable alternative to allopurinol in the event of severe AEs. It is eliminated through the liver and excreted in urine and feces, making dose adjustment unnecessary in patients with creatinine clearance ≥ 30 ml/min. Febuxostat, which is a XOI, has been shown to improve glomerular hemodynamics and reduce decline in renal function through its anti-inflammatory properties which inhibit renal vasoconstriction, preserve afferent arteriolar morphology, and reduce tubulointerstitial nephritis [50, 51].

Of note, rasburicase was not associated with a reduction in SU or composite renal events. This evidence was based on the findings reported from a single study, which had variable baseline characteristics for eGFR, diabetes mellitus, established coronary artery disease, and age between the rasburicase and placebo groups. Exclusion of this study in a sensitivity analysis on the basis that it was the only in-patient study of 5 days’ duration, indicated that lowering SU was associated with reduced composite renal events and slower decline in renal function.

To offer some clinical context to our findings, this review highlights the renoprotective benefits of allopurinol and febuxostat in asymptomatic patients with hyperuricemia without excess AEs. However, this review did not include data from populations with high incidence of severe allopurinol hypersensitivity reactions, such as Korean, Han Chinese, and Thai [2]. The incidence of severe hypersensitivity reported in the included studies was quite low and therefore the data could not be pooled, so the results should be considered cautiously in the context of these high-risk groups, which would normally require HLA-B*5801 testing prior to the use of allopurinol.

Our study has several strengths. First, this is the first quantitative review in a large number of asymptomatic hyperuricemia patients from 23 RCTs, which were included according to clearly defined inclusion and exclusion criteria following a registered protocol. Second, the NMA enabled the comparison of a wide range of all available ULTs. Third, this review considered multiple relevant outcomes including composite renal events, MACE, SU, eGFR, SBP, and AEs. Finally, this NMA of RCTs showed no inconsistency and little evidence of publication bias. Nevertheless, this review also had some limitations. Subgroup analysis by incidence of severe allopurinol hypersensitivity could not be undertaken due to the limited number of studies with available data. Composite renal and MACE outcomes were used due to the small number of reported events, which may vary in clinical significance, while some studies may not have reported symptoms of gout, stones, and tophi, weak network structure, heterogeneous of study design, the obvious heterogeneity of the study samples, e.g., comorbidity and gender distribution, and wide variety of study duration from 0.5 month to 84.0 months.

In a real-world setting, practitioners are unable to fully control residual confounding in observational studies and the effects of lowering SU on reducing renal decline and prevention of MACE is limited to a few studies [52–55] that were unable to estimate the relative effects for each individual ULT. Larger studies comparing multiple ULTs of sufficient duration are necessary.

## Conclusions

In conclusion, evidence suggests that allopurinol and febuxostat are ULTs that offer the greatest potential benefit to minimize composite renal events and improve renal function without significant risk of increased AEs.

## Supplementary Information

**Additional file 1.** 

## Data Availability

Study protocol: Available at www.crd.york.ac.uk/prospero. Statistical code and data set: Available on reasonable request from Dr. Sapankaew (e-mail, tunlanut.sap@mahidol.edu).

## Abbreviations

AEs: Adverse events
CI: Confidence interval
CKD: Chronic kidney disease
CVD: Cardiovascular disease
eGFR: Estimated glomerular filtration rate
MACE: Major adverse cardiovascular events
MD: Mean difference
NMA: Network meta-analysis
PRISMA: Preferred Reporting Items for Systematic Reviews and Meta-Analyses
PROSPERO: International Prospective Register of Systematic Reviews
RCT: Randomized controlled trial
RoB 2: A revised Cochrane risk-of-bias tool for randomized trials
RR: Risk ratio
SBP: Systolic blood pressure
SD: Standard deviation
SU: Serum urate level
SUCRA: Surface under the cumulative ranking curve
ULT: Urate-lowering therapy
XOI: Xanthine oxidase inhibitor
