# Supplementary material for: Efficacy and safety of urate-lowering agents in asymptomatic hyperuricemia: systematic review and network meta-analysis of randomized controlled trials
Source: BMC Nephrol. 2022 Jun 23;23:223. doi: 10.1186/s12882-022-02850-3 (PMC9229855; doi:10.1186/s12882-022-02850-3)

**Additional File**

**Efficacy of urate-lowering therapy agents in asymptomatic hyperuricemia: systematic review and network meta-analysis of randomized controlled trials**

| **Legends for Supplementary Tables and Figures** | **Page** |
| --- | --- |
| **Table S1.** General search terms | 1 |
| **Table S2.** Excluded studies | 2 |
| **Table S3.** Risk of bias of the included studies by RoB 2: a revised Cochrane risk-of-bias tool for randomized trials | 8 |
| **Table S4.** Description of heterogeneity and publication bias assessments for pairwise meta-analysis | 9 |
| **Table S5.** Description of heterogeneity exploration by adjustment of covariates in meta-regression | 10 |
| **Table S6.** Mixed relative treatment effects on estimated glomerular filtration rates by dosage | 12 |
| **Table S7.** Mixed relative treatment effects on serum urate level | 13 |
| **Table S8.** Mixed relative treatment effects on systolic blood pressure | 14 |
| **Table S9.** Mixed relative treatment effects on systolic blood pressure by dosage | 15 |
| **Table S10.** Sensitivity analysis excluding rasburicase: mixed relative treatment effects on composite renal events | 16 |
| **Table S11.** Sensitivity analysis excluding rasburicase: mixed relative treatment effects on serum urate level | 17 |
| **Table S12.** Surface under the cumulative ranking curve for highest efficacy and lowest adverse events of urate-lowering agents. | 18 |
| **Table S13.** Confidence in Network Meta-Analysis | 19 |
| **Figure S1.** Forest plots of composite renal events | 22 |
| **Figure S2.** Forest plots of major adverse cardiovascular events | 23 |
| **Figure S3.** Forest plots of serum urate level | 24 |
| **Figure S4.** Forest plots of estimated glomerular filtration rate | 26 |
| **Figure S5.** Forest plots of systolic blood pressure | 28 |
| **Figure S6.** Forest plots of adverse events | 30 |
| **Figure S7.** Forest plots: subgroup analysis of serum urate level by proportion of patients with hypertension | 32 |
| **Figure S8.** Forest plots: sensitivity analysis of serum urate level excluding study containing all hypertensive patients. | 33 |
| **Figure S9.** Network maps of primary outcomes | 34 |
| **Figure S10.** Network maps of secondary outcomes | 35 |
| **Figure S11.** Comparison-adjusted funnel plots | 36 |
| **Figure S12.** Scatter plot of the surface under the cumulative ranking curve for highest efficacy and lowest adverse events of urate-lowering agents | 37 |
| **Figure S13.** Network forest plot | 38 |

**Table S1.** General search terms

| **Population** | **Intervention/ Comparator** | **Surrogate Outcome** | | **Clinical Outcome** | |
| --- | --- | --- | --- | --- | --- |
| Hyperuricemia  Hyperuricaemia | Allopurinol  Febuxostat  Topiroxostat  Benzbromarone  Probenecid  Sulfinpyrazone  Rasburicase  Pegloticase  Lesinurad  Anakinra  Canakinumab  Urate lower*  Urate-lower* | *Uric acid blood level*  Uric  Urate  *Kidney function*  “glomerular filtration rate”  Creatinine  Proteinuria  *Albuminuria  Microalbuminuria  *Blood sugar*  “Blood glucose”  “Blood sugar”  “Plasma glucose”  “Glucose blood”  “Hemoglobin a1c”  “Glycosylated hemoglobin”  Hba1c | *Blood pressure*  “Blood pressure”  hypertension  *Endothelial function*  “Endothelial function”  “Endothelial *function”  “Flow mediated”  Flow-mediated  “Homeostatic model”  Homa* | *Cardiovascular disease* “Cardiovascular disease*”  “Cerebrovascular disease*”  “Cerebrovascular accident” stroke  *Heart*  “Ischemic heart” “coronary heart” “coronary artery disease*” “acute coronary”  *Arrhythmia*  “Heart failure” “Cardiac failure”  “Peripheral vascular disease*”  “peripheral artery disease” | *Diabetes mellitus*  “Diabetes mellitus”  “Diabetic nephropath*”  “Diabetic kidney”  “Diabetic neuropath*”  “Diabetic peripheral neuropath*”  “Diabetic retinopath*”  *Kidney stone*  “Kidney stone*”  “Nephrolithiasis”  “renal stone”  “renal calcu*”  *Adverse event*  “Adverse drug”  “Adverse event*”  “Drug allerg*”  “Drug hypersensitivity*” |

**Table S2.** Excluded studies

| **Not met study population** | Feig DI, Soletsky B, Johnson RJ. Effect of allopurinol on blood pressure of adolescents with newly diagnosed essential hypertension: A randomized trial. J Am Med Assoc. 2008;300(8):924-32. |
| --- | --- |
|  | Alshahawey M, Shaheen SM, Elsaid T, Sabri NA. Effect of febuxostat on oxidative stress in hemodialysis patients with endothelial dysfunction: a randomized, placebo-controlled, double-blinded study. International Urology and Nephrology. 2019 Sep;51(9):1649-57. |
|  | Wu JY, Chang YT, Lin YC, Lee CH, Loh EW, Wu MY, et al. Efficacy and Safety of Lesinurad in Patients with Hyperuricemia Associated with Gout: A Systematic Review and Meta-Analysis of Randomized Controlled Trials. Pharmacotherapy. 2018;38(11):1106-19. |
|  | Song GG, Lee YH. Comparative efficacy and safety of lesinurad 200 mg and 400 mg combined with a xanthine oxidase inhibitor in hyperuricemic patients with gout: A Bayesian network meta-analysis of randomized controlled trials. Int J Clin Pharmacol Ther. 2019;57(7):345-52. |
|  | Shi Y, Chen W, Jalal D, Li Z, Chen W, Mao H, et al. Clinical outcome of hyperuricemia in IgA nephropathy: A retrospective cohort study and randomized controlled trial. Kidney Blood Press Res. 2012;35(3):153-60. |
|  | Chen JH, Lan JL, Cheng CF, Liang WM, Lin HY, Tsay GJ, et al. Effect of urate-lowering therapy on all-cause and cardiovascular mortality in hyperuricemic patients without gout: A case-matched cohort study. PLoS ONE. 2015;10(12). |
|  | Matzkies F. Lasting normalisation of uric acid levels after treatment of patients with gout and hyperuricaemia with a combination of 300 mg allopurinol and 60 mg benzbromarone. Med Klin. 1992;87(9):460-2. |
|  | Becker MA, Schumacher HR, Jr., Wortmann RL, MacDonald PA, Palo WA, Eustace D, et al. Febuxostat, a novel nonpurine selective inhibitor of xanthine oxidase: a twenty-eight-day, multicenter, phase II, randomized, double-blind, placebo-controlled, dose-response clinical trial examining safety and efficacy in patients with gout. Arthritis Rheum. 2005;52(3):916-23. |
|  | Jomori T, Saitoh K, Hosoya T. Effects of topiroxostat on the serum urate levels and urinary albumin excretion in concomitant therapy or in primary disease of chronic kidney disease: -A post hoc analysis of multicenter, randomized, placebo-controlled, double-blind study in hyperuricemic stage 3 chronic kidney disease patients with or without gout. Jpn Pharmacol Ther. 2015;43(1):39-50. |
|  | Schumacher HR, Jr., Becker MA, Wortmann RL, Macdonald PA, Hunt B, Streit J, et al. Effects of febuxostat versus allopurinol and placebo in reducing serum urate in subjects with hyperuricemia and gout: a 28-week, phase III, randomized, double-blind, parallel-group trial. Arthritis Rheum. 2008;59(11):1540-8. |
|  | So M, Maeda T, Kashiwagi N, Takada M, Ishikawa T, Sato Y, et al. Safety and efficacy of topiroxostat for hyperuricemic patients with or without gout. Ther Res. 2017;38(8):793-804. |
|  | Hosoya T, Ohno I, Nomura S, Hisatome I, Uchida S, Fujimori S, et al. Effects of topiroxostat on the serum urate levels and urinary albumin excretion in hyperuricemic stage 3 chronic kidney disease patients with or without gout. Clin Exp Nephrol. 2014;18(6):876-84. |
|  | Terkeltaub R, Malamet R, Bos K, Li J, Goldfarb DS, Pillinger M, et al. Renal safety of lesinurad: A pooled analysis of phase III and extension studies. Ann Rheum Dis. 2017;76:379-80. |
|  | Akhondzadeh S, Milajerdi MR, Amini H, Tehrani-Doost M. Allopurinol as an adjunct to lithium and haloperidol for treatment of patients with acute mania: A double-blind, randomized, placebo-controlled trial. Bipolar Disorders. 2006;8(5 I):485-9. |
|  | Siu YP, Leung KT, Tong MK, Kwan TH. Use of allopurinol in slowing the progression of renal disease through its ability to lower serum uric acid level. American journal of kidney diseases : the official journal of the National Kidney Foundation. 2006;47(1):51-9. |
|  | Kanbay M, Ozkara A, Selcoki Y, Isik B, Turgut F, Bavbek N, et al. Effect of treatment of hyperuricemia with allopurinol on blood pressure, creatinine clearence, and proteinuria in patients with normal renal functions. Int Urol Nephrol. 2007;39(4):1227-33. |
|  | Allopurinol reduces blood pressure in adolescents with mild hypertension. Nat Clin Pract Nephrol. 2008;4(12):644-5. |
|  | Hernandez-Divers SJ, Martinez-Jimenez D, Bush S, Latimer KS, Zwart P, Kroeze EJBV. Effects of allopurinol on plasma uric acid levels in normouricaemic and hyperuricaemic green iguanas (Iguana iguana). Vet Rec. 2008;162(4):112-5. |
|  | Schumacher HR, Jr., Becker MA, Wortmann RL, Macdonald PA, Hunt B, Streit J, et al. Effects of febuxostat versus allopurinol and placebo in reducing serum urate in subjects with hyperuricemia and gout: a 28-week, phase III, randomized, double-blind, parallel-group trial. Arthritis Rheum. 2008;59(11):1540-8. |
|  | Wu AH, Ghali JK, Neuberg GW, O'Connor CM, Carson PE, Levy WC. Uric acid level and allopurinol use as risk markers of mortality and morbidity in systolic heart failure. Am Heart J. 2010;160(5):928-33. |
|  | Yiǧiner O, Özçelik F, Aparci M, Işilak Z, Uz O. The beneficial effects of allopurinol in cardiology practice: Decrease in uric acid and vascular oxidative stress/ the effects of lowering uric acid levels using allopurinol on markers of metabolic syndrome in end-stage renal disease patients: A pilot study. Anadolu Kardiyoloji Derg. 2010;10(3):294-5. |
| **Only abstract available** | McLean, L., L. Gunamardhana, U. Thienel, J. Wu, and G. Smithson. "FRI0241 The effect of febuxostat on inflammatory and cardiovascular biomarkers in hyperuricemic hypertensive patients." (2018): 661-661. |
|  | Ivanov D, Ivanova M, Bevzenko T. Febuxostat improves GFR and BP in non-diabetic adults with CKD 2-3: 6 years treatment and follow-up. InNephrology Dialysis Transplantation 2018 May 1 (Vol. 33). GREAT CLARENDON ST, OXFORD OX2 6DP, ENGLAND: OXFORD UNIV PRESS. |
|  | De Mier VP, Vergara N, Santamaria R, Buendia P, Martinez-Moreno J, Jimenez-Moral M, Muñoz-Castañeda J, Rodriguez J, Aljama P. Effect of allopurinol on endothelial function, blood pressure, arterial stiffness and renal damage in patients with chronic kidney disease and asymptomatic hyperuricemia. Journal of Hypertension. 2018 Jun 1;36:e184. |
|  | Santamaria R, Buendia P, De Mier VP, Martinez-Moreno J, Vergara N, Jimenez-Moral M, Muñoz-Castañeda J, Rodriguez J, Aljama P. EFFECT OF TREATMENT WITH ALLOPURINOL ON MARKERS OF MICROINFLAMMATION AND VASCULAR DAMAGE AND REPAIR IN PATIENTS WITH CHRONIC KIDNEY DISEASE AND ASYMPTOMATIC HYPERURICEMIA. Journal of Hypertension. 2018 Jun 1;36:e121. |
|  | Stack A, Dronamraju N, Parkinson J, Johansson S, Johnsson E, Erlandsson F, Terkeltaub R. SP262 THE IMPACT OF URATE-LOWERING DRUGS VERINURAD AND FEBUXOSTAT ON ALBUMINURIA IN TYPE 2 DIABETES. Nephrology Dialysis Transplantation. 2019 Jun 1;34(Supplement_1):gfz103-SP262. |
|  | Gaffo, Angelo, David Calhoun, Elizabeth Rahn, Suzanne Oparil, Paul Muntner, L. I. Peng, David Redden et al. "OP0208 EFFECT OF SERUM URATE LOWERING WITH ALLOPURINOL ON BLOOD PRESSURE IN YOUNG ADULTS." (2019): 180-180. |
|  | Arakawa J, Ayaori M, Nishida T, Sasaki M, Komatsu T, Suenaga Y, Nita R, Uto-Kondo H, Sakurada M, Ikewaki K. A Xanthine Oxidase Inhibitor Febuxostat Improves Endothelial Function as Evaluated by Flow-Mediated Vasodilatation in Hyperuricemic Patients. Circulation. 2018 Nov 6;138(Suppl_1):A14447-. |
|  | Ogino K, Kinugasa Y, Kato M, Yamamoto K, Hamada T, Hisatome I. Uric-acid lowering treatment by a xanthine oxidase inhibitor improved the diastolic function in patients with hyperuricemia. Journal of Cardiac Failure. 2019 Aug 1;25(8):S26. |
|  | Nagaraju SP, Attur RP, Rangaswamy D, Rao I, Rao SP, Kaza S, Shenoy S, Saraf K, Bhojaraja M, Ramaswamy A, Koulmane Laxminarayana SL. SP296 effect of febuxostat versus allopurinol on hyperuricemia and progression of chronic kidney disease. Nephrology Dialysis Transplantation. 2017 May 1;32(suppl_3):iii205-6. |
|  | Suzuki S, Yoshihisa A, Sato A, Shimizu T, Sato T, Sakamoto N, Nakazato K, Tsuda T, Tsuda A, Goto J, Ishibashi T. Multicenter Randomized Controlled Trial Between Febuxostat and Allopurinol in Chronic Heart Failure Patients With Hyperuricemia. Circulation. 2017 Nov 14;136(suppl_1):A14509-. |
| **On urate lowering agents prior to enrollment** | Ohta Y, Ishizuka A, Arima H, Hayashi S, Iwashima Y, Kishida M, Yoshihara F, Nakamura S, Kawano Y. Effective uric acid-lowering treatment for hypertensive patients with hyperuricemia. Hypertension Research. 2017 Mar;40(3):259-63. |
|  | Mizukoshi T, Kato S, Ando M, Sobajima H, Ohashi N, Naruse T, et al. Renoprotective effects of topiroxostat for Hyperuricaemic patients with overt diabetic nephropathy study (ETUDE study): A prospective, randomized, multicentre clinical trial. Nephrology. 2018;23(11):1023-30. |
|  | Hanvivadhanakul P, Akkasilpa S, Deesomchok U. Efficacy of benzbromarone compared to allopurinol in lowering serum uric acid level in hyperuricemic patients. J Med Assoc Thailand. 2002;85(SUPPL. 1):S40-S7. |
|  | Akkasilpa S, Osiri M, Deesomchok U, Avihingsanon Y. The efficacy of combined low dose of allopurinol and benzbromarone compared to standard dose of allopurinol in hyperuricemia. J Med Assoc Thailand. 2004;87(9):1087-91. |
|  | Reinders MK, van Roon EN, Houtman PM, Brouwers JRBJ, Jansen TLTA. Biochemical effectiveness of allopurinol and allopurinol-probenecid in previously benzbromarone-treated gout patients. Clin Rheumatol. 2007;26(9):1459-65. |
|  | Bantia S, Harman L, Parker C, Papac D, Maetzel A, Taubenheim B, et al. Ulodesine (BCX4208) add-on therapy to allopurinol 300mg lowers hypoxanthine and xanthine plasma levels in a dose-dependent fashion: Results from a 12-week randomized controlled trial in patients with gout. Arthritis Rheum. 2012;64:S698. |
|  | Suzuki S, Yoshihisa A, Sato A, Shimizu T, Sato T, Sakamoto N, et al. Multicenter randomized controlled trial between febuxostat and allopurinol in chronic heart failure patients with hyperuricemia. Circulation. 2017;136. |
|  | Bardin T, Karra RG, So A, Tausche AK, Wild I, Hagedorn H, et al. Lesinurad (LESU) adjunctive therapy with allopurinol (ALLO) in patients not responding to allo monotherapy: Pooled post hoc safety and efficacy analysis in a patient subgroup using concomitant diuretics at baseline (BL). Ann Rheum Dis. 2018;77:1050-1. |
| **No data for pooling** | Gunawardhana L, McLean L, Punzi HA, Hunt B, Palmer RN, Whelton A, Feig DI. Effect of febuxostat on ambulatory blood pressure in subjects with hyperuricemia and hypertension: a phase 2 randomized placebo‐controlled study. Journal of the American Heart Association. 2017 Nov 4;6(11):e006683. |
|  | Ibrahim SE, Helmi A, Yousef TM, Hassan MS, Farouk N. Association of asymptomatic hyperuricemia and endothelial dysfunction in psoriatic arthritis. Egypt Rheumatol. 2012;34(2):83-9. |
|  | Walter-Sack I, de Vries JX, Ernst B, Frei M, Kolb S, Kosmowski J, Priebe U, Schroder HE, Slotty C, Voss A, Weber A. Uric acid lowering effect of oxipurinol sodium in hyperuricemic patients-therapeutic equivalence to allopurinol. The Journal of Rheumatology. 1996 Mar 1;23(3):498-501. |
|  | Mertz DP, Eichhorn R. Does benzbromarone in therapeutic doses raise renal excretion of oxipurinol?. Klinische Wochenschrift. 1984 Dec;62(24):1170-2. |
|  |  |
|  | Wu TH, Chen LC, Yang LL. Hypouricemic effect and regulatory effects on autonomic function of Shao-Yao Gan-Cao Tang, a Chinese herbal prescription, in asymptomatic hyperuricemic vegetarians. Rheumatol Int. 2007;28(1):27-31. |
|  | A Randomised Study of Allopurinol on Endothelial Function and eGFR in Asymptomatic Hyperuricemic Subjects with Normal Renal Function(Clinical Journal of the American Society of Nephrology, (August 2011), 8, (887-894),doi: 10.2215/CJN.10801011). Clin J Am Soc Nephrol. 2011;6(12):2901-2. |
|  | Shiramoto M, Sugeno M, Liu S, Shen Z, Hall J. Pharmacodynamics, pharmacokinetics, and safety of verinurad in combination with febuxostat versus febuxostat alone and verinurad alone in japanese adults with gout or asymptomatic hyperuricemia: A phase 2a open-label study. Arthritis Rheum. 2016;68:252-3. |
|  | Huang Y, Meng J, Sun B, Xiang T, Zhou X, Xu B, et al. Acupuncture for serum uric acid in patients with asymptomatic hyperuricemia: A randomized, double-blind, placebo-controlled trial. Int J Cardiol. 2017;232:227-32. |
| **Observational studies** | Levy G, Shi JM, Cheetham TC, Rashid N. Urate-lowering therapy in moderate to severe chronic kidney disease. The Permanente Journal. 2018;22. |
|  | Pai BS, Swarnalatha G, Ram R, Dakshinamurty KV. Allopurinol for prevention of progression of kidney disease with hyperuricemia. Indian Journal of Nephrology. 2013 Jul;23(4):280. |
|  | Song K, Wang Y, Wang G, Zhang Q, Jiao H, Huang G, Lu W. Does decreasing serum uric acid level prevent hypertension?–a nested RCT in cohort study: rationale, methods, and baseline characteristics of study cohort. BMC public health. 2013 Dec;13(1):1-7. |
|  | Bayram D, Tuğrul Sezer M, Inal S, Altuntaş A, Kıdır V, Orhan H. The effects of allopurinol on metabolic acidosis and endothelial functions in chronic kidney disease patients. Clinical and experimental nephrology. 2015 Jun;19(3):443-9. |
| **Ongoing studies** | Sakuma M, Toyoda S, Arikawa T, Koyabu Y, Kato T, Adachi T, Suwa H, Narita JI, Anraku K, Ishimura K, Yamauchi F. The effects of xanthine oxidase inhibitor in patients with chronic heart failure complicated with hyperuricemia: a prospective randomized controlled clinical trial of topiroxostat vs allopurinol—study protocol. Clinical and experimental nephrology. 2018 Dec;22(6):1379-86. |
|  | Yokota T, Fukushima A, Kinugawa S, Okumura T, Murohara T, Tsutsui H. Randomized Trial of Effect of Urate-Lowering Agent Febuxostat in Chronic Heart Failure Patients with Hyperuricemia (LEAF-CHF) Study Design. International heart journal. 2018:17-560. |
|  | Hosoya T, Kimura K, Itoh S, Inaba M, Uchida S, Tomino Y, et al. The effect of febuxostat to prevent a further reduction in renal function of patients with hyperuricemia who have never had gout and are complicated by chronic kidney disease stage 3: Study protocol for a multicenter randomized controlled study. Trials. 2014;15(1). |
|  | Collantes E, Anon J, Tinahones F, Sanchez P. Lipid profile associated with asymptomatic hyperuricemia. Protocolized study of 77 subjects. REV ESP REUMATOL. 1989;16(5):129-31. |
|  | Sakuma M, Toyoda S, Arikawa T, Koyabu Y, Kato T, Adachi T, et al. The effects of xanthine oxidase inhibitor in patients with chronic heart failure complicated with hyperuricemia: a prospective randomized controlled clinical trial of topiroxostat vs allopurinol—study protocol. Clin Exp Nephrol. 2018;22(6):1379-86. |
| **Single arm studies** | Ferreira M, Jiménez C, Lopez MO, González E, Santana MJ, Selgas R. Short-term efficacy and safety of treatment with febuxostat in kidney transplant recipient. An unicentric observational study. Nefrología (English Edition). 2018 May 1;38(3):331-2. |
|  | Meléndez-Ramírez G, Pérez-Méndez O, López-Osorio C, Kuri-Alfaro J, Espinola-Zavaleta N. Effect of the treatment with allopurinol on the endothelial function in patients with hyperuricemia. Endocrine research. 2012 Feb 1;37(1):1-6. |
|  | Nishizawa T, Taniura T, Nomura S. Effects of febuxostat on platelet-derived microparticles and adiponectin in patients with hyperuricema. Blood Coagulation & Fibrinolysis. 2015 Dec;26(8):887. |
|  | Yelken B, Caliskan Y, Gorgulu N, Altun I, Yilmaz A, Yazici H, Oflaz H, Yildiz A. Reduction of uric acid levels with allopurinol treatment improves endothelial function in patients with chronic kidney disease. Clinical nephrology. 2012 Apr 1;77(4):275-82. |

**Table S3.** Risk of bias of the included studies by RoB 2: a revised Cochrane risk-of-bias tool for randomized trials

| **Authors** | **Year** | **Randomization process** | **Deviations from intended interventions** | **Missing outcome data** | **Measurement of the outcome** | **Selection of the reported result** | **Overall** |
| --- | --- | --- | --- | --- | --- | --- | --- |
| Doehner | 2002 |  |  |  |  |  |  |
| Siu | 2006 |  |  |  |  |  |  |
| Ogino | 2009 |  |  |  |  |  |  |
| Kanbay | 2011 |  |  |  |  |  |  |
| Ejaz | 2012 |  |  |  |  |  |  |
| Jalalzadeh | 2012 |  |  |  |  |  |  |
| Taheraghdam | 2014 |  |  |  |  |  |  |
| Goicoechea | 2015 |  |  |  |  |  |  |
| Liu | 2015 |  |  |  |  |  |  |
| Sircar | 2015 |  |  |  |  |  |  |
| Takir | 2015 |  |  |  |  |  |  |
| Nakagomi | 2015 |  |  |  |  |  |  |
| Sezai | 2015 |  |  |  |  |  |  |
| Tani | 2015 |  |  |  |  |  |  |
| Tsurata | 2015 |  |  |  |  |  |  |
| Beddhu | 2016 |  |  |  |  |  |  |
| Kojima | 2016 |  |  |  |  |  |  |
| Golmohammadi | 2017 |  |  |  |  |  |  |
| McMullan | 2017 |  |  |  |  |  |  |
| Jalal | 2018 |  |  |  |  |  |  |
| Kimura | 2018 |  |  |  |  |  |  |
| Mukri | 2018 |  |  |  |  |  |  |
| Kojima | 2019 |  |  |  |  |  |  |
|  |  |  |  |  |  |  |  |
|  |  |  | low  risk |  | some concerns |  | high risk |

**Table S4.** Description of heterogeneity and publication bias assessments for pairwise meta-analysis

| **Comparison** | **Composite renal events** | | **Major adverse cardiovascular events** | | **Serum urate level** | | **Estimated glomerular filtration rate** | | **Systolic blood pressure** | | **Adverse events** | |
| --- | --- | --- | --- | --- | --- | --- | --- | --- | --- | --- | --- | --- |
|  | **I^2^ (%)** | **Egger’s test *P*-value** | **I^2^ (%)** | **Egger’s test *P*-value** | **I^2^ (%)** | **Egger’s test *P*-value** | **I^2^ (%)** | **Egger’s test *P*-value** | **I^2^ (%)** | **Egger’s test *P*-value** | **I^2^ (%)** | **Egger’s test *P*-value** |
| Allopurinol vs placebo/no ULT | 38.2 | 0.3 | 0.0 | - | 98.7 | 0.7 | 0.0 | 1.0 | 83.1 | 0.4 | 25.7 | 0.2 |
| Febuxostat vs placebo/no ULT | 0.0 | 0.7 | 28.2 | 0.7 | 91.1 | 0.2 | 19.5 | 0.8 | 0.0 | 0.0 | 71.4 | 0.0 |
| Febuxostat vs allopurinol | - | - | 0.0 | - | 99.4 | 0.2 | 0.0 | 0.3 | 62.4 | - | 58.5 | - |

ULT, urate-lowering therapy.

**Table S5.** Description of heterogeneity exploration by adjustment of covariates in meta-regression

| **Outcome/ Comparison** | **None** | | **Duration** | | **Age** | | **%M** | | **BMI** | |
| --- | --- | --- | --- | --- | --- | --- | --- | --- | --- | --- |
|  | **Studies** | **I^2^ (%)** | **Studies** | **I^2^ (%)** | **Studies** | **I^2^ (%)** | **Studies** | **I^2^ (%)** | **Studies** | **I^2^ (%)** |
| Composite renal events  Allopurinol vs placebo/no ULT | 3 | 80.8 | 3 | 89.3 | 3 | 88.7 | - | - | - | - |
| MACE  Febuxostat vs placebo/no ULT | 3 | 74.1 | 3 | 77.2 | 3 | 86.9 | 3 | 29.9 | - | - |
| SU Allopurinol vs placebo/no ULT | 9 | 95.3 | 9 | 95.1 | 8 | 94.5 | 8 | 95.7 | 6 | 93.6 |
| SU  Febuxostat vs placebo/no ULT | 6 | 91.1 | 6 | 79.3 | 6 | 92.8 | 6 | 92.6 | 4 | 95.5 |
| SU  Febuxostat vs allopurinol/no ULT | 3 | 99.4 | 3 | 99.3 | 3 | 93.2 | 3 | 99.2 | - | - |
| SBP Allopurinol vs placebo/no ULT | 7 | 82.9 | 7 | 85.6 | 7 | 85.7 | 6 | 87.3 | 5 | 88.5 |
| AEs Allopurinol vs placebo/no ULT | 6 | 94.6 | 6 | 93.5 | 6 | 95.7 | 5 | 96.6 | 3 | 96.0 |
| AEs  Febuxostat vs placebo/no ULT | 3 | 98.7 | 3 | 97.7 | 3 | 99.3 | 3 | 0.0 | - | - |

| **Outcome/ Comparison** | **% HT** | | **% DM** | | **% CKD** | | **% CVD** | | **Dose** | |
| --- | --- | --- | --- | --- | --- | --- | --- | --- | --- | --- |
|  | **Studies** | **I^2^ (%)** | **Studies** | **I^2^ (%)** | **Studies** | **I^2^ (%)** | **Studies** | **I^2^ (%)** | **Studies** | **I^2^ (%)** |
| Composite renal events Allopurinol vs placebo/no ULT | - | - | - | - | - | - | - | - | 3 | 85.7 |
| MACE  Febuxostat vs placebo/no ULT | 3 | 86.7 | - | - | 3 | 74.1 | 3 | 86.9 | 3 | 86.6 |
| SU Allopurinol vs placebo/no ULT | 3 | 88.4 | 3 | 98.6 | 4 | 97.3 | 3 | 95.6 | 8 | 95.3 |
| SU  Febuxostat vs placebo/no ULT | 6 | 27.6 | 5 | 93.9 | 6 | 81.5 | 6 | 89.2 | 6 | 90.4 |
| SU  Febuxostat vs allopurinol/no ULT | 3 | 99.1 | 3 | 99.3 | - | - | - | - | - | - |
| SBP Allopurinol vs placebo/no ULT | - | - | - | - | 4 | 89.3 | - | - | 7 | 84.1 |
| AEs Allopurinol vs placebo/no ULT | - | - | 3 | 65.4 | 3 | 57.5 | 3 | 0.0 | 6 | 94.5 |
| AEs  Febuxostat vs placebo/no ULT | 3 | 99.3 | - | - | 3 | 98.7 | 3 | 98.9 | 3 | 98.7 |

AEs, adverse events; BMI, body mass index; CKD, chronic kidney disease; CVD, cardiovascular disease; DM, diabetes mellitus; HT, hypertension; M, male; MACE, major adverse cardiovascular events; SBP, systolic blood pressure; SU, serum urate level; ULT, urate-lowering therapy.

**Table S6.** Mixed relative treatment effects on estimated glomerular filtration rates by dosage

| **placebo /no ULT** | **-4.38  (-7.25, -1.52)** | -0.50  (-9.72, 8.72) | -2.19  (-6.61, 2.24) | **-3.08  (-5.51, -0.66)** |
| --- | --- | --- | --- | --- |
| **4.38  (1.52 ,7.25)** | **low-dose allopurinol** | 3.88  (-5.77, 13.54) | 2.19  (-2.52, 6.90) | 1.30  (-1.28, 3.88) |
| 0.50  (-8.72 ,9.72) | -3.88  (-13.54, 5.77) | **low-dose febuxostat** | -1.69  (-11.91, 8.54) | -2.58  (-12.12, 6.95) |
| 2.19  (-2.24, 6.61) | -2.19  (-6.90, 2.52) | 1.69  (-8.54, 11.91) | **high-dose allopurinol** | -0.90  (-5.02, 3.23) |
| **3.08  (0.66, 5.51)** | -1.30  (-3.88, 1.28) | 2.58  (-6.95, 12.12) | 0.90  (-3.23, 5.02) | **high-dose febuxostat** |

ULT, urate-lowering therapy.

Testing of inconsistency, χ^2^ = 4.33, *P*-value = 0.1145. Results are mean differences (95% confidence intervals) between each pair of treatments from network meta-analysis. Comparisons are read counterclockwise. Low-dose allopurinol was defined as < 300 mg/day, high-dose allopurinol as ≥ 300 mg/day, low-dose febuxostat as < 40 mg/day, and high-dose febuxostat as ≥ 40 mg/day.

**Table S7.** Mixed relative treatment effects on serum urate level

| **placebo /no ULT** | **-1.63**  **(-2.51, -0.74)** | **-2.45**  **(-3.85, -1.04)** | **-2.49**  **(-3.66, -1.31)** | **-4.30**  **(-6.32, -2.27)** | **-2.45**  **(-3.21, -1.70)** | **-3.29**  **(-4.07, -2.51)** |
| --- | --- | --- | --- | --- | --- | --- |
| **1.63**  **(0.74, 2.51)** | **low-dose allopurinol** | -0.82  (-2.48, 0.84) | -0.86  (-2.31, 0.59) | **-2.67**  **(-4.88, -0.46)** | -0.83  (-1.96, 0.30) | **-1.66**  **(-2.73, -0.60)** |
| **2.45**  **(1.04, 3.85)** | 0.82  (-0.84, 2.48) | **low-dose febuxostat** | -0.04  (-1.87, 1.79) | -1.85  (-4.31, 0.62) | -0.01  (-1.60, 1.59) | -0.84  (-2.45, 0.76) |
| **2.49**  **(1.31, 3.66)** | 0.86  (-0.59, 2.31) | 0.04  (-1.79, 1.87) | **uricosuric agents** | -1.81  (-4.15, 0.53) | 0.03  (-1.13, 1.19) | -0.80  (-2.15, 0.54) |
| **4.30**  **(2.27, 6.32)** | **2.67**  **(0.46, 4.88)** | 1.85  (-0.62, 4.31) | 1.81  (-0.53, 4.15) | **rasburicase** | 1.84  (-0.32, 4.00) | 1.01  (-1.17, 3.18) |
| **2.45**  **(1.70, 3.21)** | 0.83  (-0.30, 1.96) | 0.01  (-1.59, 1.60) | -0.03  (-1.19, 1.13) | -1.84  (-4.00, 0.32) | **high-dose allopurinol** | -0.83  (-1.75, 0.08) |
| **3.29**  **(2.51, 4.07)** | **1.66**  **(0.60, 2.73)** | 0.84  (-0.76, 2.45) | 0.80  (-0.54, 2.15) | -1.01  (-3.18, 1.17) | 0.83  (-0.08, 1.75) | **high-dose febuxostat** |

ULT, urate-lowering therapy.

Testing of inconsistency, χ^2^ = 1.39, *P*-value = 0.92. Results are mean differences (95% confidence intervals) between each pair of treatments from network meta-analysis. Comparisons are read counterclockwise. Low-dose allopurinol was defined as < 300 mg/day, high-dose allopurinol as ≥ 300 mg/day, low-dose febuxostat as < 40 mg/day, and high-dose febuxostat as ≥ 40 mg/day. Uricosuric agents consist of probenecid and benzbromarone.

**Table S8.** Mixed relative treatment effects on systolic blood pressure

| **placebo /no ULT** | -3.45  (-9.07, 2.17) | -2.23  (-8.90, 4.45) | 0.18  (-12.81, 13.17) |
| --- | --- | --- | --- |
| 3.45  (-2.17, 9.07) | **allopurinol** | 1.22  (-6.13, 8.57) | 3.63  (-9.37, 16.62) |
| 2.23  (-4.45, 8.90) | -1.22  (-8.57, 6.13) | **febuxostat** | 2.41  (-11.82, 16.63) |
| -0.18  (-13.17, 12.81) | -3.63  (-16.62, 9.37) | -2.41  (-16.63, 11.82) | **probenecid** |

ULT, urate-lowering therapy.

Testing of inconsistency, χ^2^ = 2.12, *P*-value = 0.35. Results are mean differences (95% confidence intervals) between each pair of treatments from network meta-analysis. Comparisons are read counterclockwise.

**Table S9.** Mixed relative treatment effects on systolic blood pressure by dosage

| **placebo /no ULT** | 9.50  (-0.29, 19.29) | -1.52  (-15.14, 12.10) | -2.19  (-24.01, 19.64) | 1.39  (-11.64, 14.42) | 4.87  (-17.30, 27.03) |
| --- | --- | --- | --- | --- | --- |
| -9.50  (-19.29, 0.29) | **low-dose allopurinol** | -11.01  (-27.80, 5.78) | -8.11  (-24.39, 8.18) | -9.39  (-25.60, 6.81) | -11.80  (-31.85, 8.26) |
| 1.52  (-12.10, 15.14) | 11.01  (-5.78, 27.80) | **low-dose febuxostat** | 2.90  (-15.94, 21.75) | 1.62  (-17.13, 20.36) | -0.78  (-22.97, 21.40) |
| 2.19  (-19.64, 24.01) | 8.11  (-8.18, 24.39) | -2.90  (-21.75, 15.94) | **probenecid** | -1.29  (-19.61, 17.03) | -3.69  (-25.51, 18.13) |
| -1.39  (-14.42, 11.64) | 9.39  (-6.81, 25.60) | -1.62  (-20.36, 17.13) | 1.29  (-17.03, 19.61) | **high-dose allopurinol** | -2.40  (-24.14, 19.33) |
| -4.87  (-27.03, 17.30) | 11.80  (-8.26, 31.85) | 0.78  (-21.40, 22.97) | 3.69  (-18.13, 25.51) | 2.40  (-19.33, 24.14) | **high-dose febuxostat** |

ULT, urate-lowering therapy.

Testing of inconsistency, χ^2^ = 0.19, *P*-value = 0.91. Results are mean differences (95% confidence intervals) between each pair of treatments from network meta-analysis. Comparisons are read counterclockwise. Low-dose allopurinol was defined as < 300 mg/day, high-dose allopurinol as ≥ 300 mg/day, low-dose febuxostat as < 40 mg/day, and high-dose febuxostat as ≥ 40 mg/day.

**Table S10.** Sensitivity analysis excluding rasburicase: mixed relative treatment effects on composite renal events

| **placebo /no ULT** | **0.39  (0.23, 0.66)** | **0.68  (0.46, 0.99)** | 0.37  (0.05, 2.69) |
| --- | --- | --- | --- |
| **2.55  (1.51, 4.30)** | **allopurinol** | 1.72  (0.94, 3.17) | 0.95  (0.14, 6.55) |
| **1.48  (1.01, 2.17)** | 0.58  (0.32, 1.07) | **febuxostat** | 0.55  (0.07, 4.09) |
| 2.68  (0.37, 19.27) | 1.05  (0.15, 7.21) | 1.81  (0.24, 13.37) | **probenecid** |

ULT, urate-lowering therapy.

Testing of inconsistency, χ^2^ = 3.16, *P*-value = 0.20. Results are relative risks (95% confidence intervals) between each pair of treatments from network meta-analysis. Comparisons are read counterclockwise.

**Table S11.** Sensitivity analysis excluding rasburicase: mixed relative treatment effects on serum urate level

| **placebo /no ULT** | **-1.63**  **(-2.51, -0.74)** | **-2.45**  **(-3.85, -1.04)** | **-2.49**  **(-3.66, -1.31)** | **-3.29**  **(-4.07, -2.51)** | **-2.45**  **(-3.21, -1.70)** |
| --- | --- | --- | --- | --- | --- |
| **1.63**  **(0.74, 2.51)** | **low-dose allopurinol** | -0.82  (-2.48, 0.84) | -0.86  (-2.31, 0.59) | **-1.66**  **(-2.73, -0.60)** | -0.83  (-1.96, 0.30) |
| **2.45**  **(1.04, 3.85)** | 0.82  (-0.84, 2.48) | **low-dose febuxostat** | -0.04  (-1.87, 1.79) | -0.84  (-2.45, 0.76) | -0.01  (-1.60, 1.59) |
| **2.49**  **(1.31, 3.66)** | 0.86  (-0.59, 2.31) | 0.04  (-1.79, 1.87) | **uricosuric**  **agents** | -0.80  (-2.15, 0.54) | 0.03  (-1.13, 1.19) |
| **3.29**  **(2.51, 4.07)** | **1.66**  **(0.60, 2.73)** | 0.84  (-0.76, 2.45) | 0.80  (-0.54, 2.15) | **high-dose febuxostat** | 0.83  (-0.08, 1.75) |
| **2.45**  **(1.70, 3.21)** | 0.83  (-0.30, 1.96) | 0.01  (-1.59, 1.60) | -0.03  (-1.19, 1.13) | -0.83  (-1.75, 0.08) | **high-dose allopurinol** |

ULT, urate-lowering therapy.

Testing of inconsistency, χ^2^ = 1.39, *P*-value = 0.92. Results are mean differences (95% confidence intervals) between each pair of treatments from network meta-analysis. Comparisons are read counterclockwise. Low-dose allopurinol was defined as < 300 mg/day, high-dose allopurinol as ≥ 300 mg/day, low-dose febuxostat as < 40 mg/day, high-dose febuxostat as ≥ 40 mg/day. Uricosuric agents consist of probenecid and benzbromarone.

**Table S12.** Surface under the cumulative ranking curve for highest efficacy and lowest adverse events of urate-lowering agents.

| **ULT** | **Adverse events** | **MACE** | **Composite renal events** | **eGFR** | **Serum urate** | **SBP** |
| --- | --- | --- | --- | --- | --- | --- |
| Placebo | 53.30 | 15.00 | 20.00 | 0.00 | 0.00 | 29.50 |
| Allopurinol | 50.00 | 50.00 | 100.00 | 85.00 | 35.00 | 74.30 |
| Febuxostat | 40.00 | 85.00 | 66.70 | 65.00 | 70.00 | 38.20 |
| Probenecid | 76.70 |  | 13.30 |  | 45.00 |  |
| Rasburicase |  |  |  |  | 100.00 |  |

eGFR; estimated glomerular filtration rates, MACE; major adverse cardiovascular events, SBP; systolic blood pressure, ULT; urate lowering therapy agents.

**Table S13.** Confidence in Network Meta-Analysis

| **Outcomes** | **Number of studies** | **Within-study bias** | **Reporting bias** | **Indirectness** | **Imprecision** | **Heterogeneity** | **Incoherence** | **Confidence rating** | **Reason(s) for downgrading** |
| --- | --- | --- | --- | --- | --- | --- | --- | --- | --- |
| **MACE** |  | | | | | | | | |
| Allopurinol vs Placebo/no-ULT | 2 | Some concerns | Some concerns | No concerns | Major concerns | No concerns | No concerns | Very low | Within-study bias,  Reporting bias, Imprecision |
| Febuxostat vs Placebo/no-ULT | 3 | Some concerns | Some concerns | No concerns | Some concerns | Some concerns | No concerns | Low | Within-study bias, Reporting bias, Imprecision, Heterogeneity |
| Allopurinol vs Febuxostat | 2 | Some concerns | Some concerns | Some concerns | Major concerns | No concerns | No concerns | Very low | Within-study bias, Reporting bias, Indirectness, Imprecision |
| **Composite renal events** |  | | | | | | | | |
| Allopurinol vs Placebo/no-ULT | 3 | Some concerns | Some concerns | No concerns | No concerns | No concerns | No concerns | Low | Within-study bias,  Reporting bias |
| Febuxostat vs Placebo/no-ULT | 3 | No concerns | Some concerns | No concerns | No concerns | Major concerns | No concerns | Very Low | Reporting bias, Heterogeneity |
| Rasburicase vs Placebo/no-ULT | 1 | No concerns | Some concerns | Some concerns | Major concerns | No concerns | No concerns | Very Low | Reporting bias, Indirectness, Imprecision |
| Allopurinol vs Febuxostat | 1 | Some concerns | Some concerns | No concerns | Major concerns | No concerns | No concerns | Very Low | Within-study bias, Reporting bias, Imprecision |
| Allopurinol vs Rasburicase | 0 | No concerns | Some concerns | No concerns | No concerns | Major concerns | No concerns | Very Low | Reporting bias, Heterogeneity |
| Febuxostat vs Rasburicase | 0 | No concerns | Some concerns | No concerns | Major concerns | No concerns | No concerns | Very Low | Reporting bias, Imprecision |
| **Serum urate levels** |  | | | | | | | | |
| Low-dose allurinol vs Placebo/no-ULT | 4 | Some concerns | Low risk | No concerns | No concerns | Some concerns | No concerns | Low | Within-study bias, Heterogeneity |
| Low-dose febuxostat vs Placebo/no-ULT | 2 | Some concerns | Low risk | No concerns | No concerns | No concerns | No concerns | Moderately | Within-study bias |
| Uricosurics vs Placebo/no-ULT | 2 | Some concerns | Low risk | No concerns | No concerns | No concerns | No concerns | Moderately | Within-study bias |
| Rasburicase vs Placebo/no-ULT | 1 | No concerns | Low risk | Some concerns | No concerns | No concerns | No concerns | Moderately | Indirectness |
| High-dose allopurinol vs Placebo/no-ULT | 5 | Some concerns | Low risk | No concerns | No concerns | No concerns | No concerns | Moderately | Within-study bias |
| High-dose febuxostat vs Placebo/no-ULT | 4 | Some concerns | Low risk | No concerns | No concerns | No concerns | No concerns | Moderately | Within-study bias |
| High-dose febuxostat vs Low-dose allopurinol | 1 | Some concerns | Low risk | No concerns | No concerns | No concerns | No concerns | Moderately | Within-study bias |
| High-dose allopurinol vs Uricosurics | 2 | Some concerns | Low risk | No concerns | No concerns | Major concerns | No concerns | Very low | Within-study bias, Heterogeneity |
| High-dose febuxostat vs High-dose allopurinol | 2 | Some concerns | Low risk | No concerns | No concerns | Some concerns | No concerns | Low | Within-study bias, Heterogeneity |
| Low-dose febuxostat vs Low-dose allopurinol | 0 | Some concerns | Low risk | No concerns | Some concerns | Some concerns | No concerns | Low | Within-study bias, Imprecision, Heterogeneity |
| Uricosurics vs Low-dose allopurinol | 0 | Some concerns | Low risk | No concerns | Some concerns | Some concerns | No concerns | Low | Within-study bias, Imprecision, Heterogeneity |
| Rasburicase vs Low-dose allopurinol | 0 | Some concerns | Low risk | No concerns | No concerns | No concerns | No concerns | Moderately | Within-study bias |
| High-dose allopurinol vs Low-dose allopurinol | 0 | Some concerns | Low risk | No concerns | No concerns | Some concerns | No concerns | Low | Within-study bias, Heterogeneity |
| Uricosurics vs Low-dose febuxostat | 0 | Some concerns | Low risk | No concerns | Major concerns | No concerns | No concerns | Very low | Within-study bias, Imprecision |
| Rasburicase vs Low-dose febuxostat | 0 | Some concerns | Low risk | No concerns | No concerns | Some concerns | No concerns | Low | Within-study bias, Heterogeneity |
| High-dose allopurinol vs Low-dose febuxostat | 0 | Some concerns | Low risk | No concerns | Major concerns | No concerns | No concerns | Very low | Within-study bias, Imprecision |
| High-dose febuxostat vs Low-dose febuxostat | 0 | Some concerns | Low risk | No concerns | Some concerns | Some concerns | No concerns | Low | Within-study bias, Imprecision, Heterogeneity |
| Rasburicase vs Uricosurics | 0 | Some concerns | Low risk | No concerns | No concerns | Some concerns | No concerns | Low | Within-study bias, Heterogeneity |
| High-dose febuxostat vs Uricosurics | 0 | Some concerns | Low risk | No concerns | Some concerns | Some concerns | No concerns | Low | Within-study bias, Imprecision, Heterogeneity |
| High-dose allopurinol vs Rasburicase | 0 | No concerns | Low risk | No concerns | No concerns | Some concerns | No concerns | Moderately | Heterogeneity |
| High-dose febuxostat vs Rasburicase | 0 | No concerns | Low risk | No concerns | Some concerns | Some concerns | No concerns | Low | Imprecision, Heterogeneity |
| **estimated Glomerular filtration rates** |  | | | | | | | | |
| Allopurinol vs Placebo/no-ULT | 4 | Some concerns | Some concerns | No concerns | No concerns | No concerns | Major concerns | Very low | Within-study bias,  Reporting bias, Incoherence |
| Febuxostat vs Placebo/no-ULT | 4 | Some concerns | Some concerns | No concerns | No concerns | Some concerns | Some concerns | Low | Within-study bias, Reporting bias, Heterogeneity, Incoherence |
| Allopurinol vs Febuxostat | 3 | Major concerns | Some concerns | No concerns | Major concerns | No concerns | Some concerns | Very low | Within-study bias, Reporting bias, Imprecision, Incoherence |
| Systolic blood pressure |  | | | | | | | | |
| Allopurinol vs Placebo/no-ULT | 7 | Some concerns | Some concerns | No concerns | Major concerns | No concerns | Some concerns | Very low | Within-study bias, Reporting bias, Imprecision, Incoherence |
| Febuxostat vs Placebo/no-ULT | 4 | Some concerns | Some concerns | No concerns | Major concerns | No concerns | Some concerns | Very low | Within-study bias, Reporting bias, Imprecision, Incoherence |
| Probenecid vs Placebo/no-ULT | 1 | Some concerns | Some concerns | No concerns | Major concerns | No concerns | No concerns | Very low | Within-study bias,  Reporting bias, Imprecision |
| Allopurinol vs Febuxostat | 2 | Some concerns | Some concerns | Some concerns | Major concerns | No concerns | No concerns | Very low | Within-study bias, Reporting bias, Indirectness, Imprecision |
| Probenecid vs Allopurinol | 1 | Some concerns | Some concerns | No concerns | Major concerns | No concerns | No concerns | Very low | Within-study bias,  Reporting bias, Imprecision |
| Probenecid vs Febuxostat | 0 | Some concerns | Some concerns | No concerns | Major concerns | No concerns | No concerns | Very low | Within-study bias,  Reporting bias, Imprecision |
| **Adverse events** |  | | | | | | | | |
| Allopurinol vs Placebo/no-ULT | 6 | Some concerns | Some concerns | No concerns | Major concerns | No concerns | No concerns | Very low | Within-study bias,  Reporting bias, Imprecision |
| Febuxostat vs Placebo/no-ULT | 3 | Some concerns | Some concerns | No concerns | Major concerns | No concerns | No concerns | Very low | Within-study bias, Reporting bias, Imprecision |
| Probenecid vs Placebo/no-ULT | 1 | Some concerns | Some concerns | No concerns | Major concerns | No concerns | No concerns | Very low | Within-study bias, Reporting bias, Imprecision |
| Allopurinol vs Febuxostat | 2 | Some concerns | Some concerns | Some concerns | Major concerns | No concerns | No concerns | Very low | Within-study bias, Reporting bias, Indirectness, Imprecision |
| Probenecid vs Allopurinol | 1 | Some concerns | Some concerns | No concerns | Major concerns | No concerns | No concerns | Very low | Within-study bias, Reporting bias, Imprecision |
| Probenecid vs Febuxostat | 0 | Some concerns | Some concerns | No concerns | Major concerns | No concerns | No concerns | Very low | Within-study bias, Reporting bias, Imprecision |

**Figure S1.** Forest plots of composite renal events

a) allopurinol vs placebo/no ULT


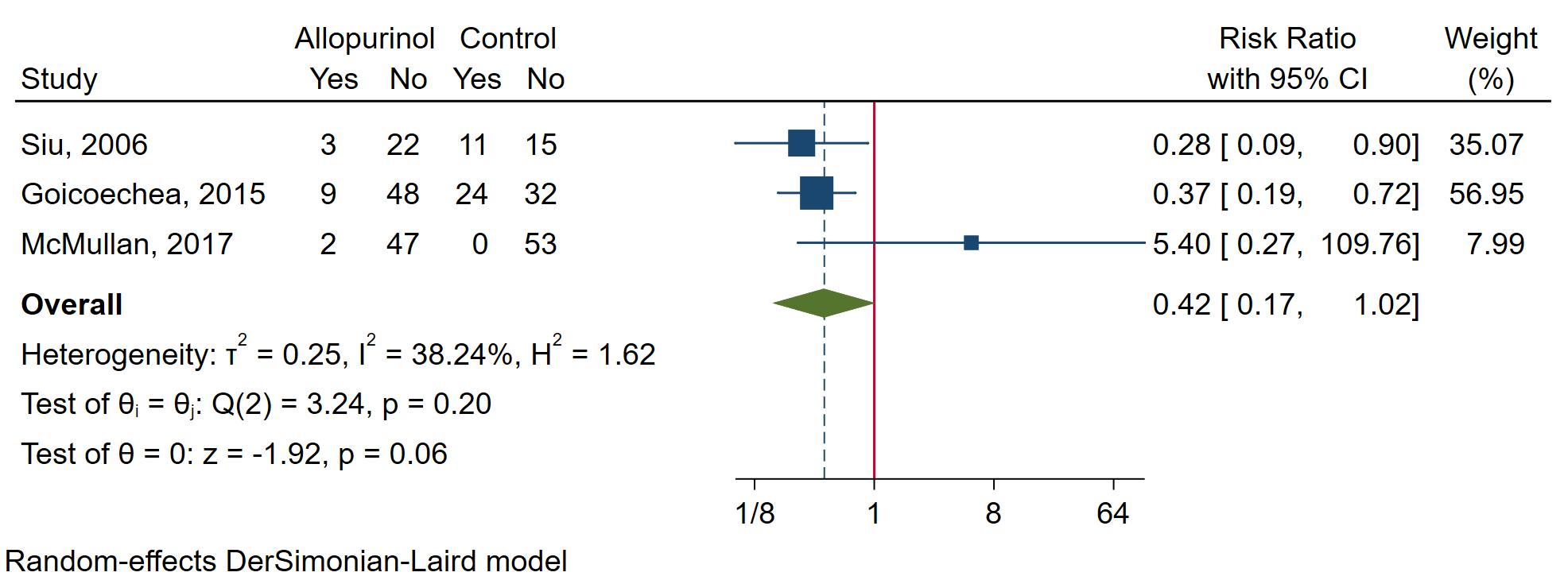


b) febuxostat vs placebo/no ULT


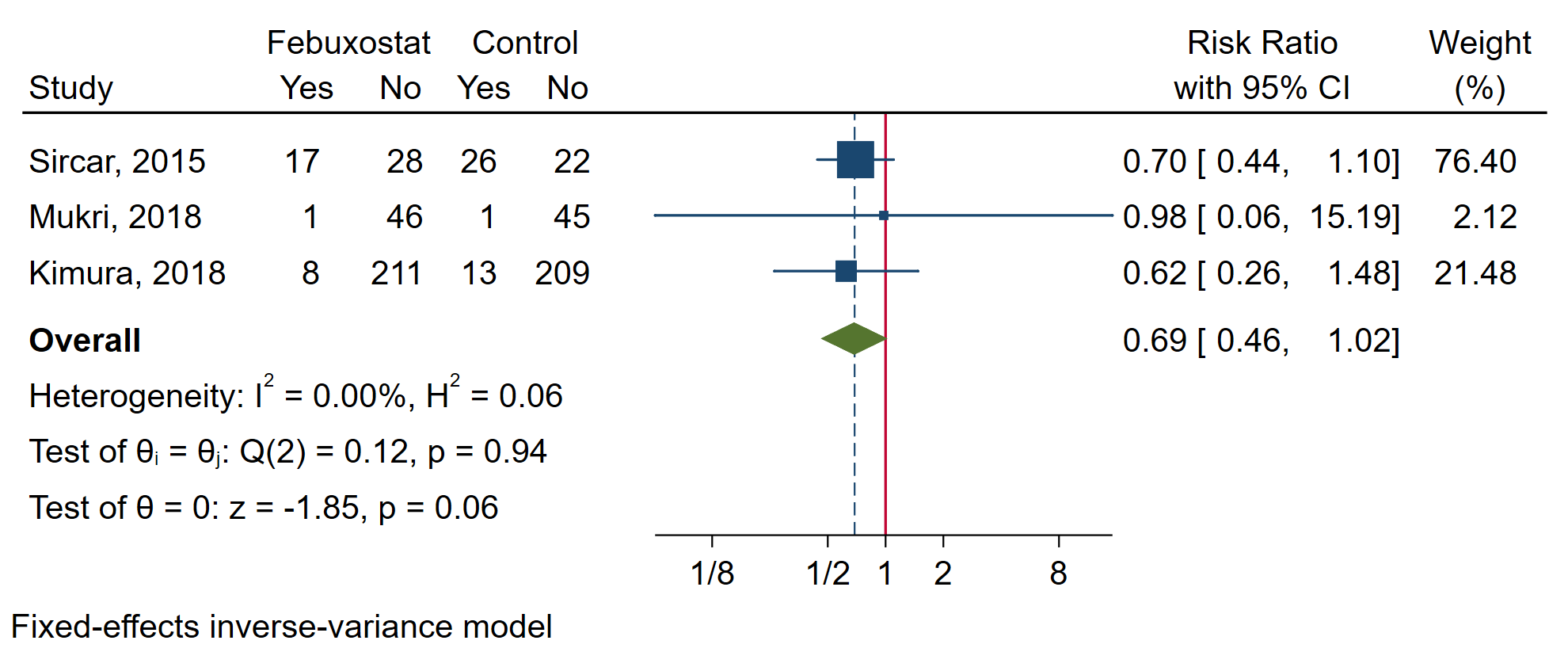


CI, confidence interval; ULT, urate-lowering therapy.

**Figure S2.** Forest plots of major adverse cardiovascular events

a) allopurinol vs placebo/no ULT


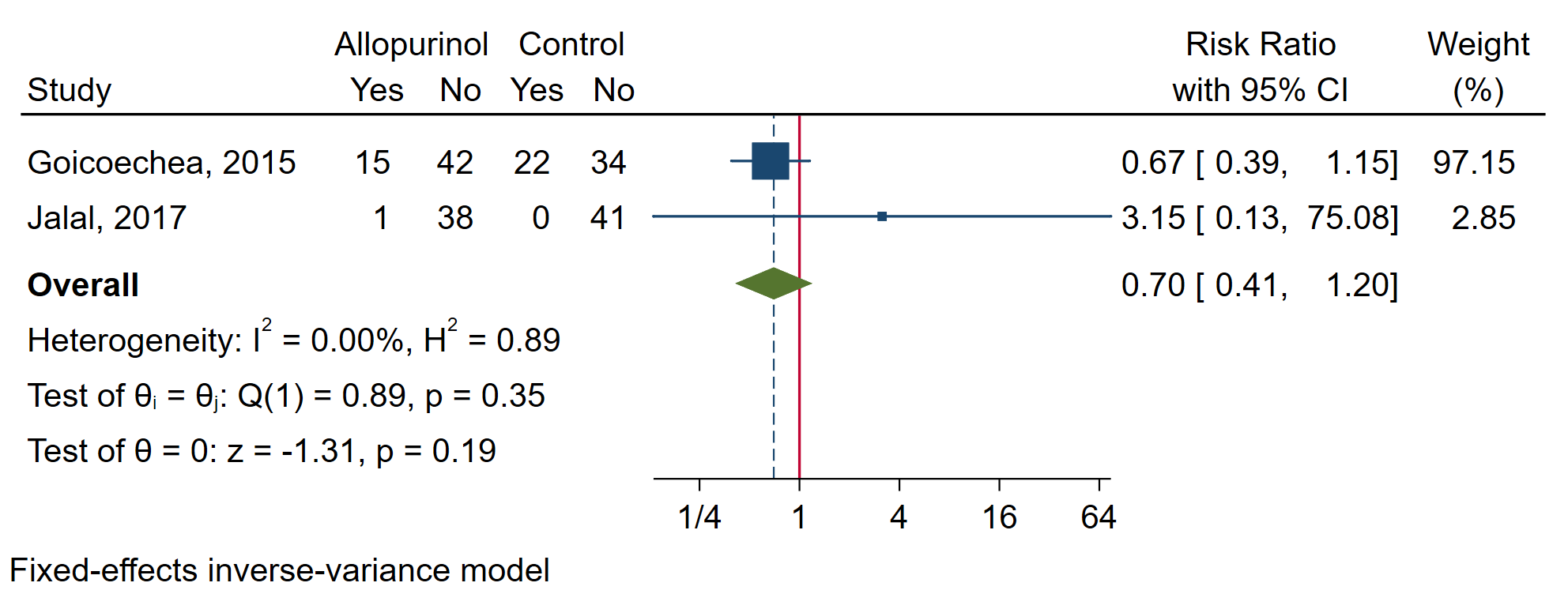


b) febuxostat vs placebo/no ULT


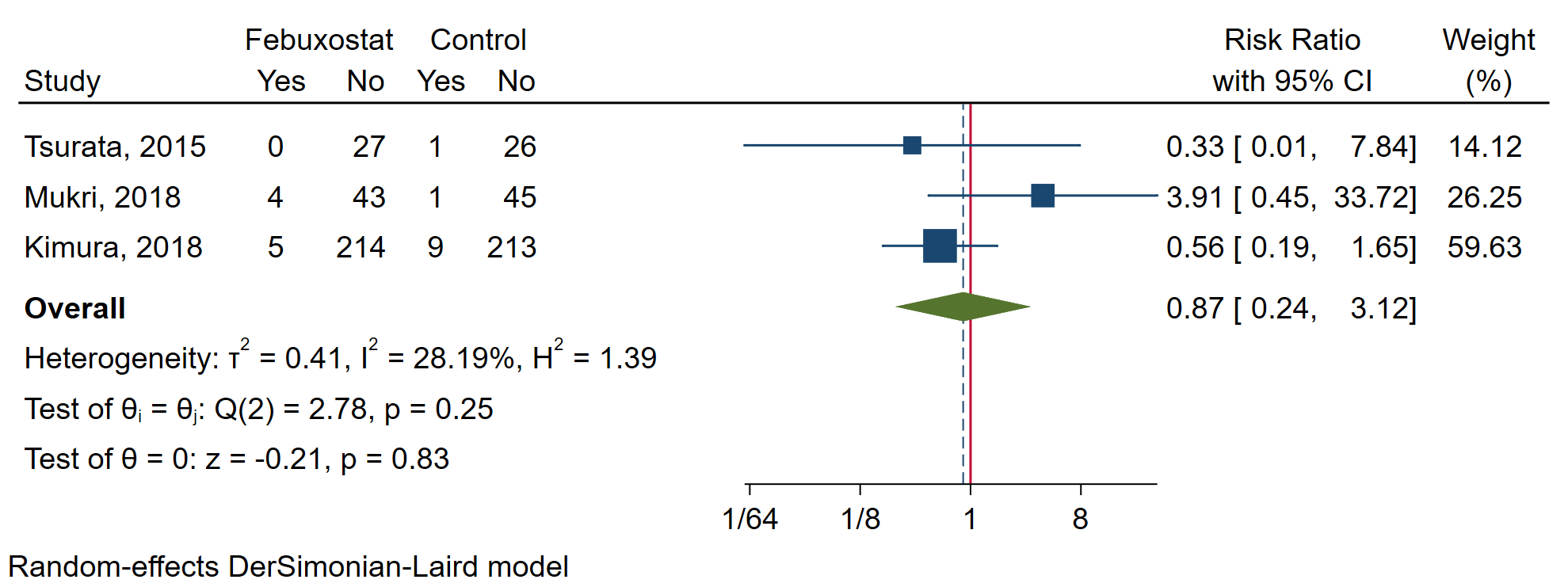


c) febuxostat vs allopurinol


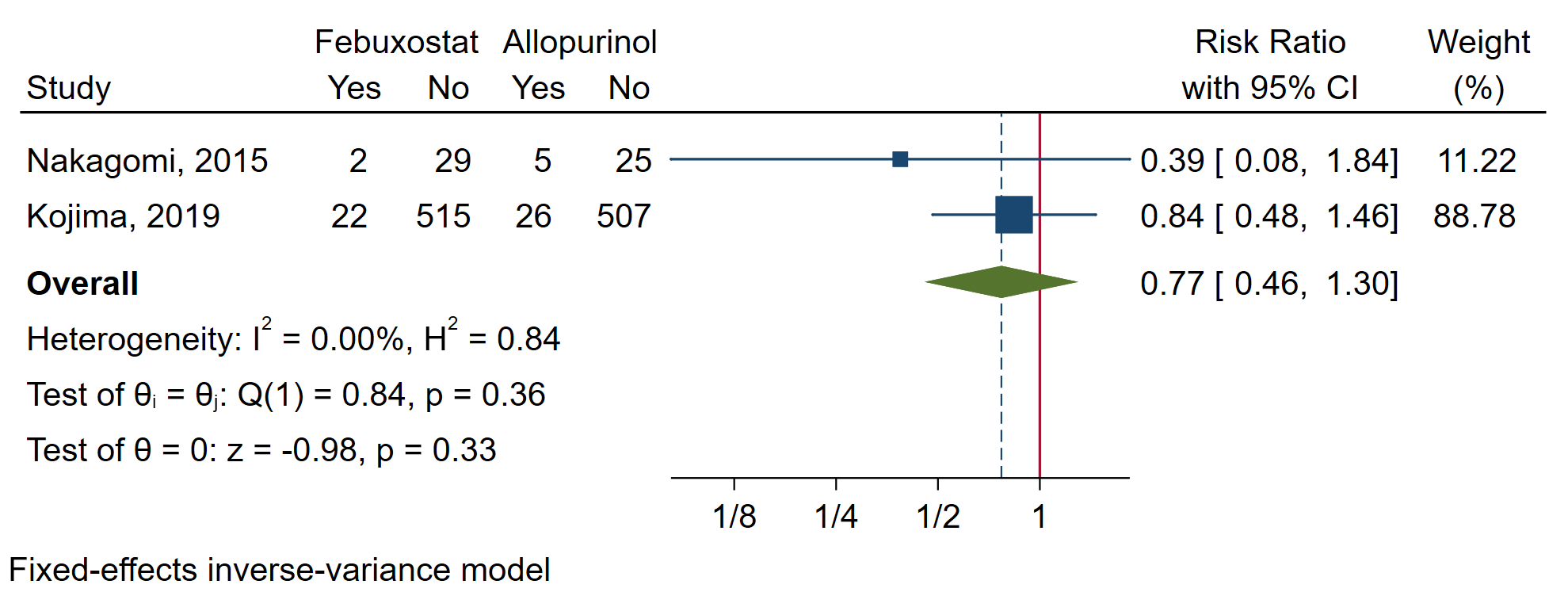


CI, confidence interval; ULT, urate-lowering therapy.

**Figure S3.** Forest plot of serum urate level

a) allopurinol vs placebo/no ULT


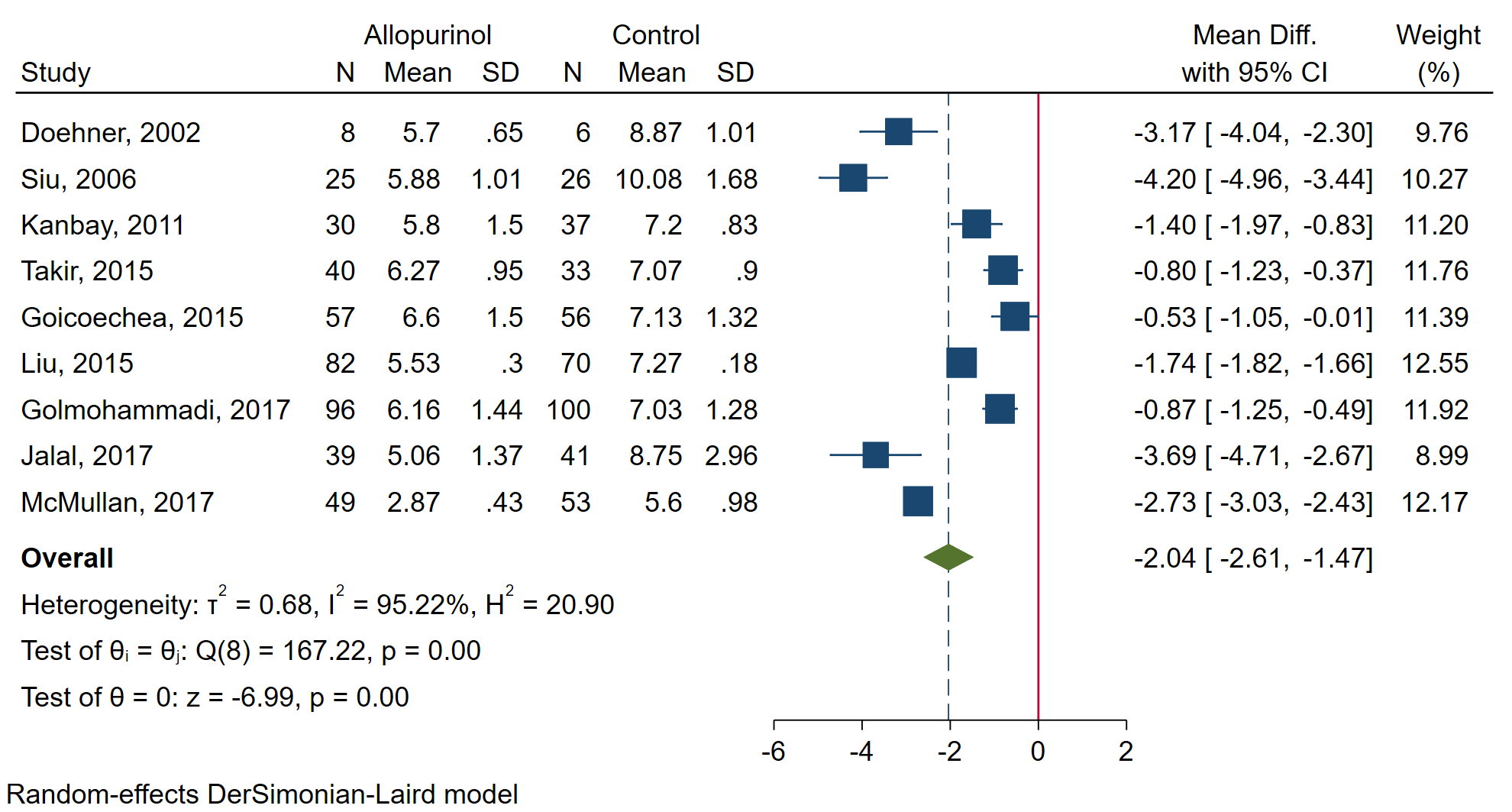


b) febuxostat vs placebo/no ULT


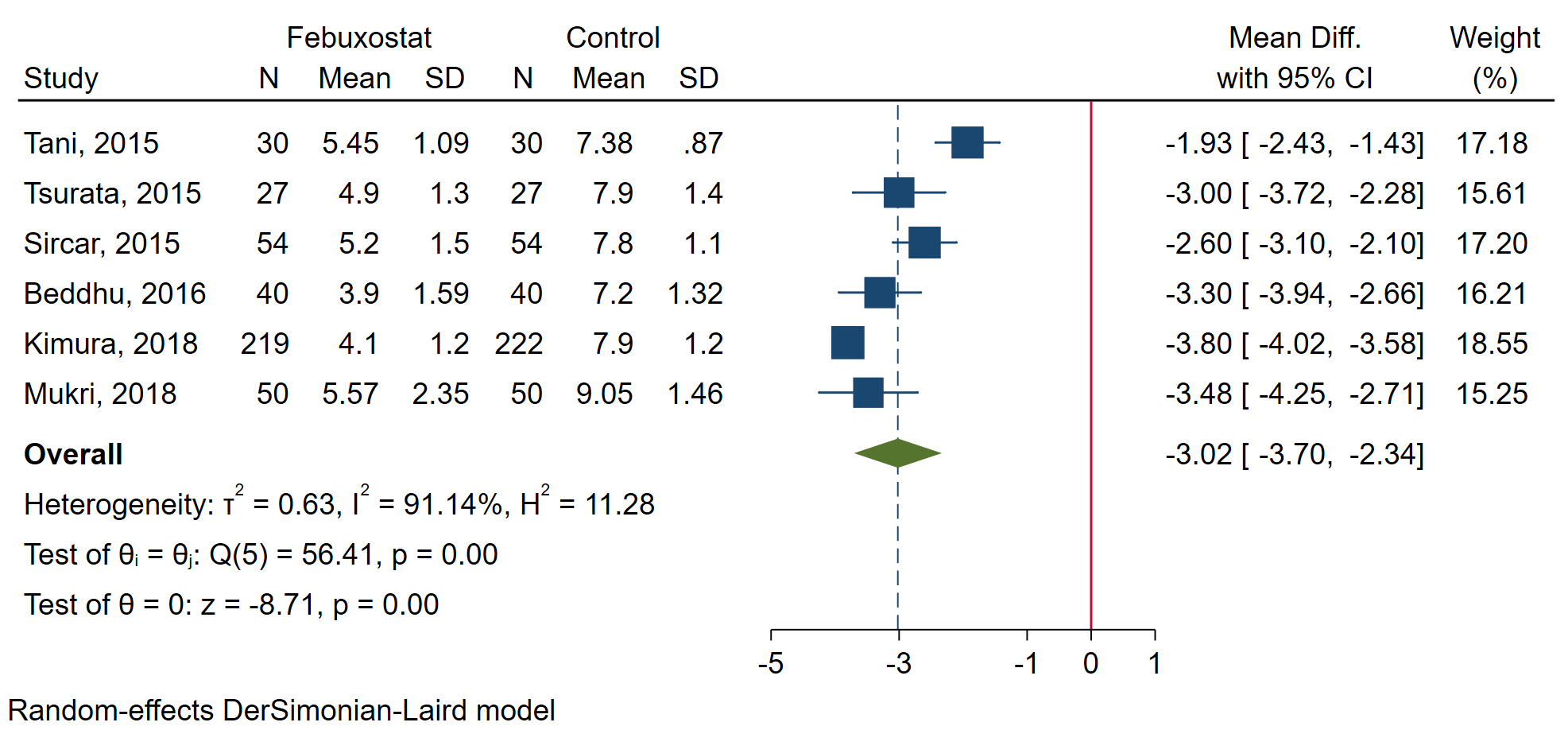


c) febuxostat vs allopurinol


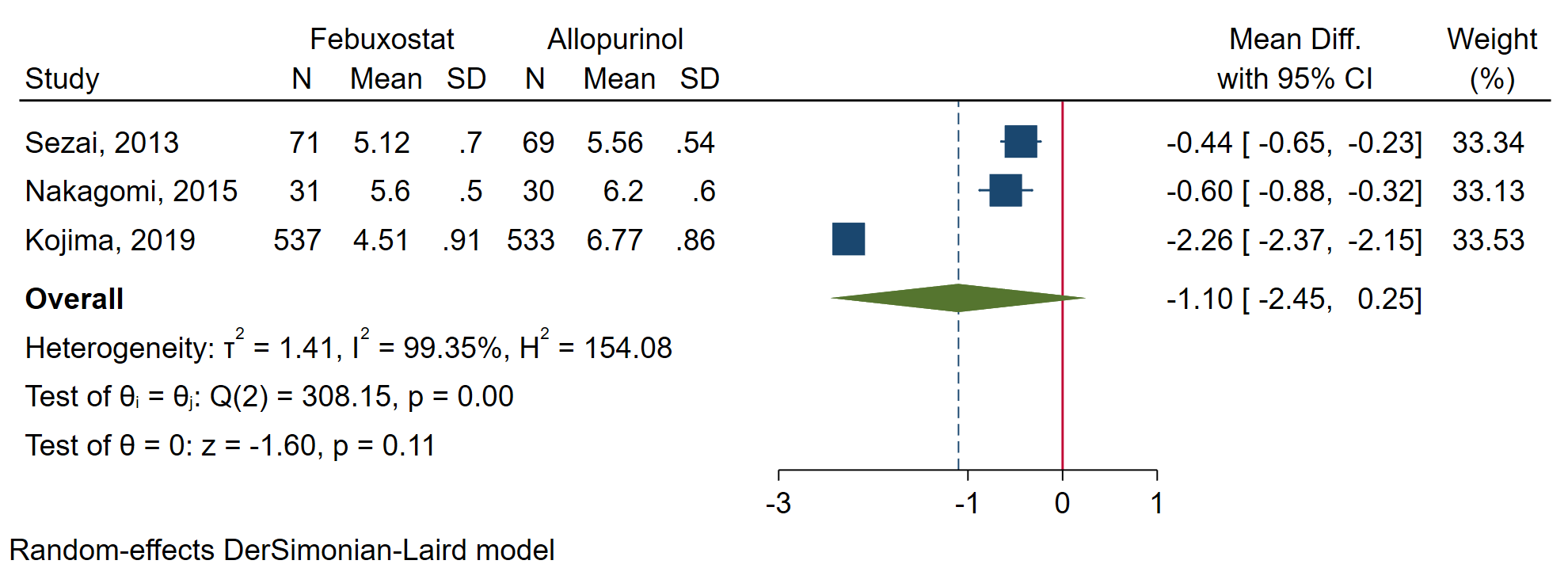


CI, confidence interval; Mean Diff., mean difference; SD, standard deviation; ULT, urate-lowering therapy.

**Figure S4.** Forest plot of estimated glomerular filtration rate

a) allopurinol vs placebo/no ULT


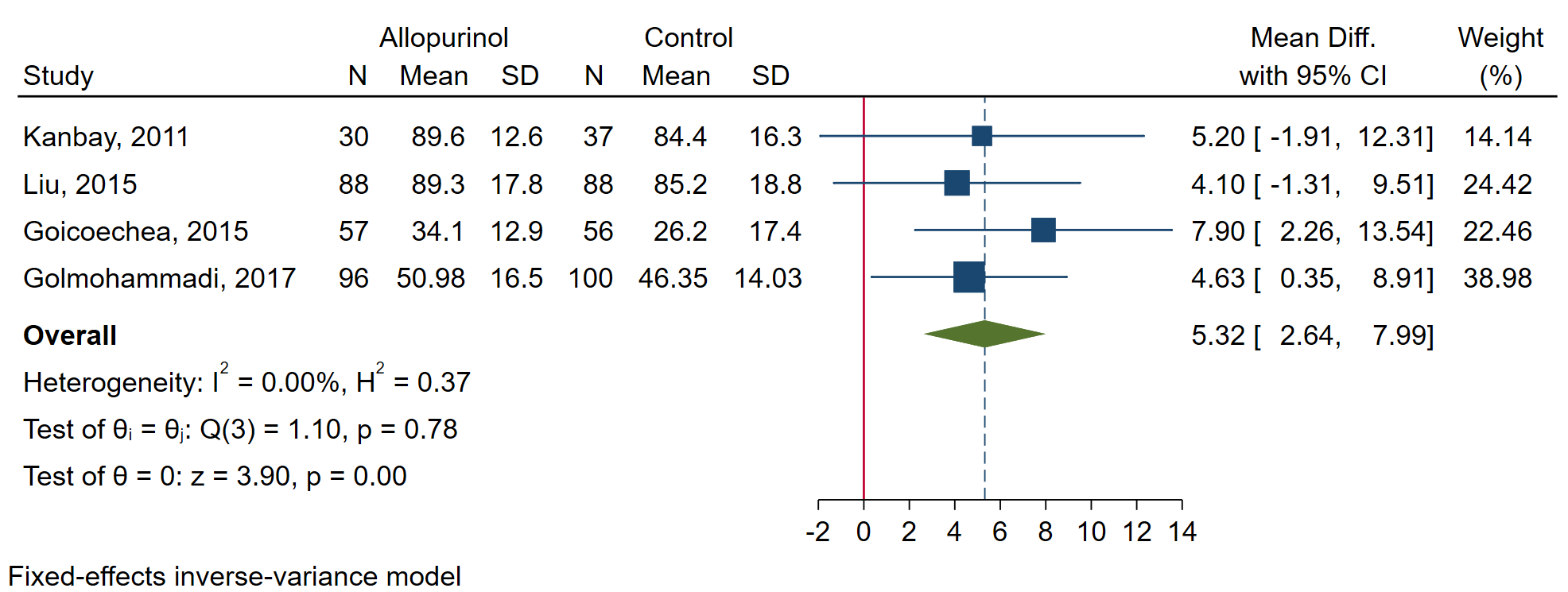


b) febuxostat vs placebo/no ULT


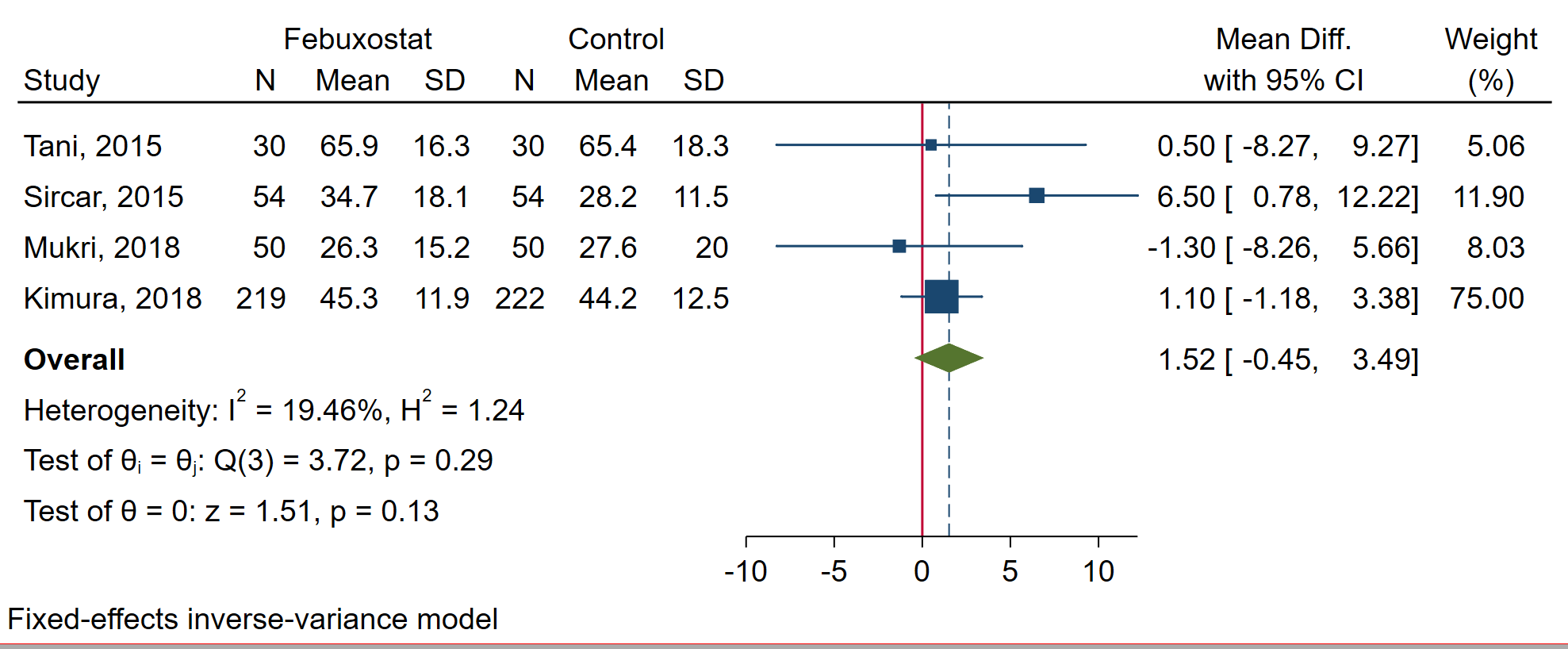


c) febuxostat vs allopurinol


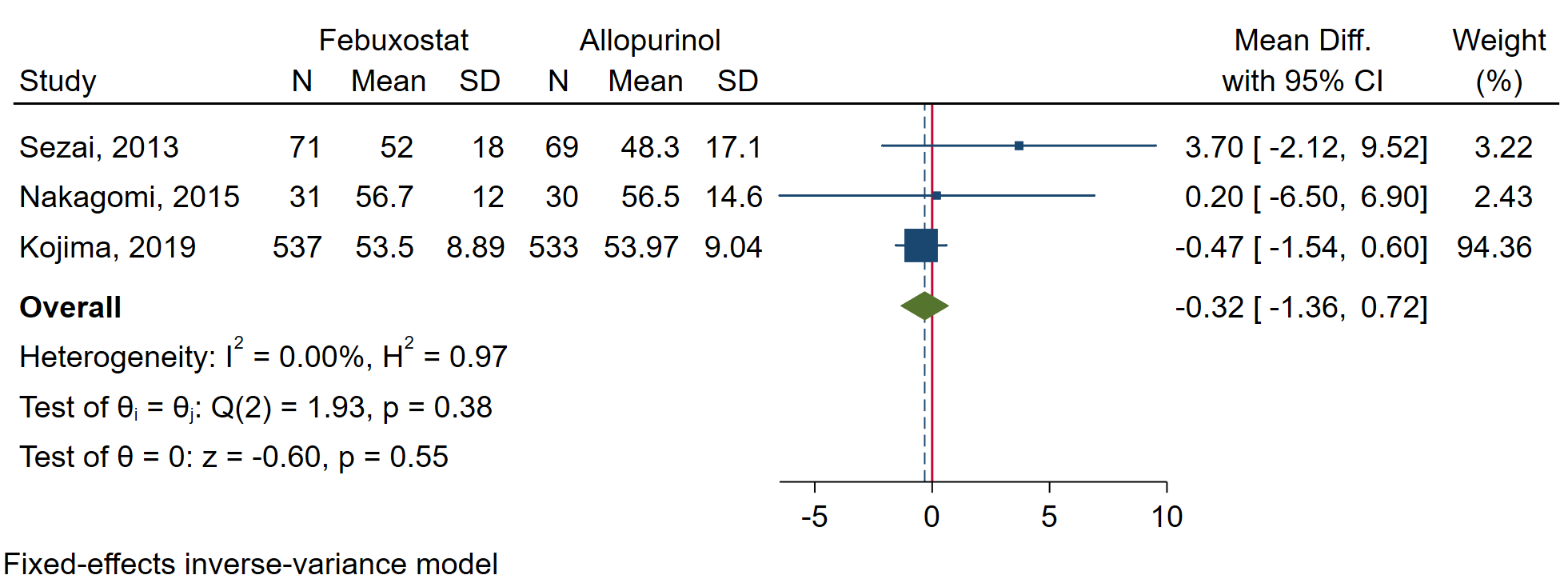


CI, confidence interval; Mean Diff., mean difference; SD, standard deviation; ULT, urate-lowering therapy.

**Figure S5.** Forest plots of systolic blood pressure

a) allopurinol vs placebo/no ULT


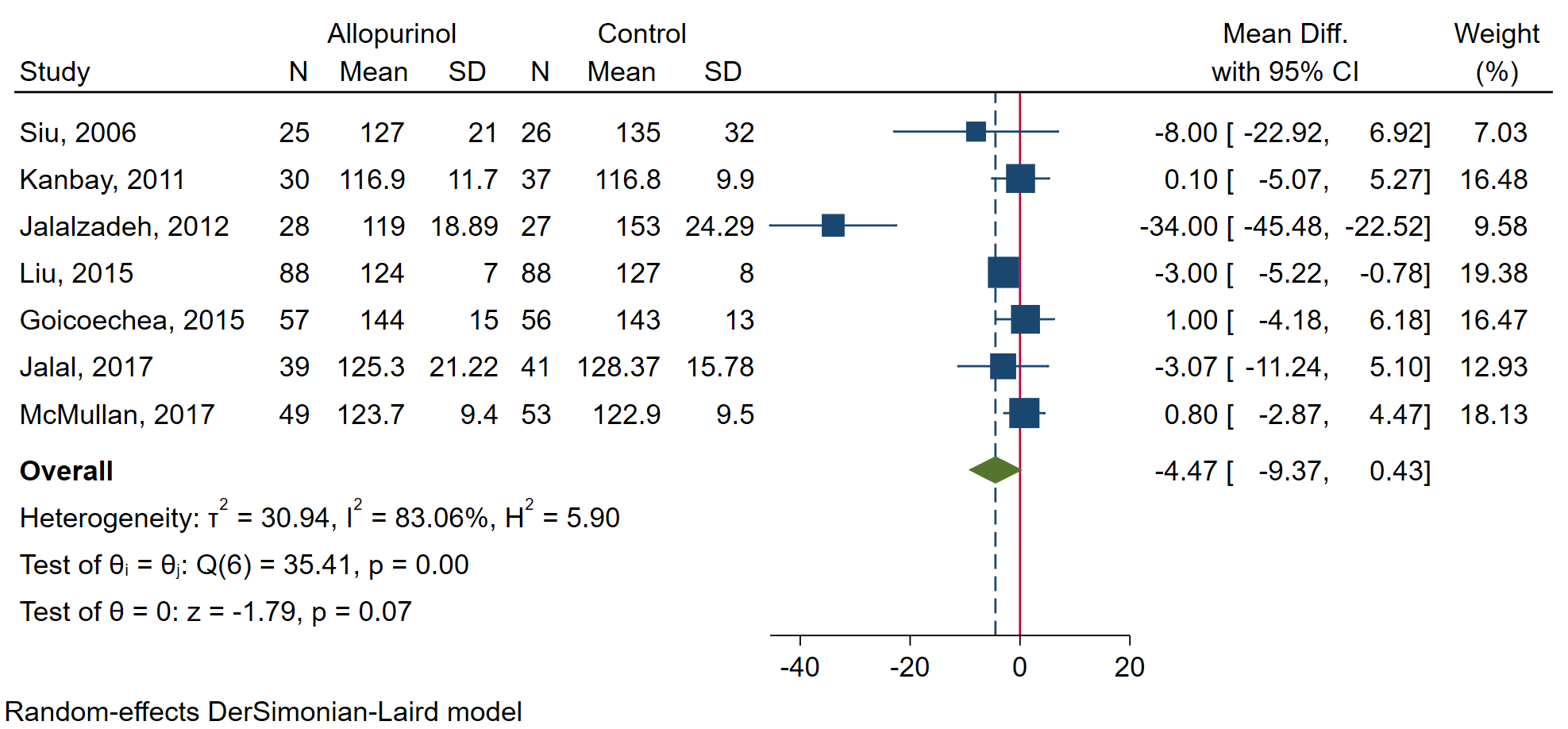


b) febuxostat vs placebo/no ULT


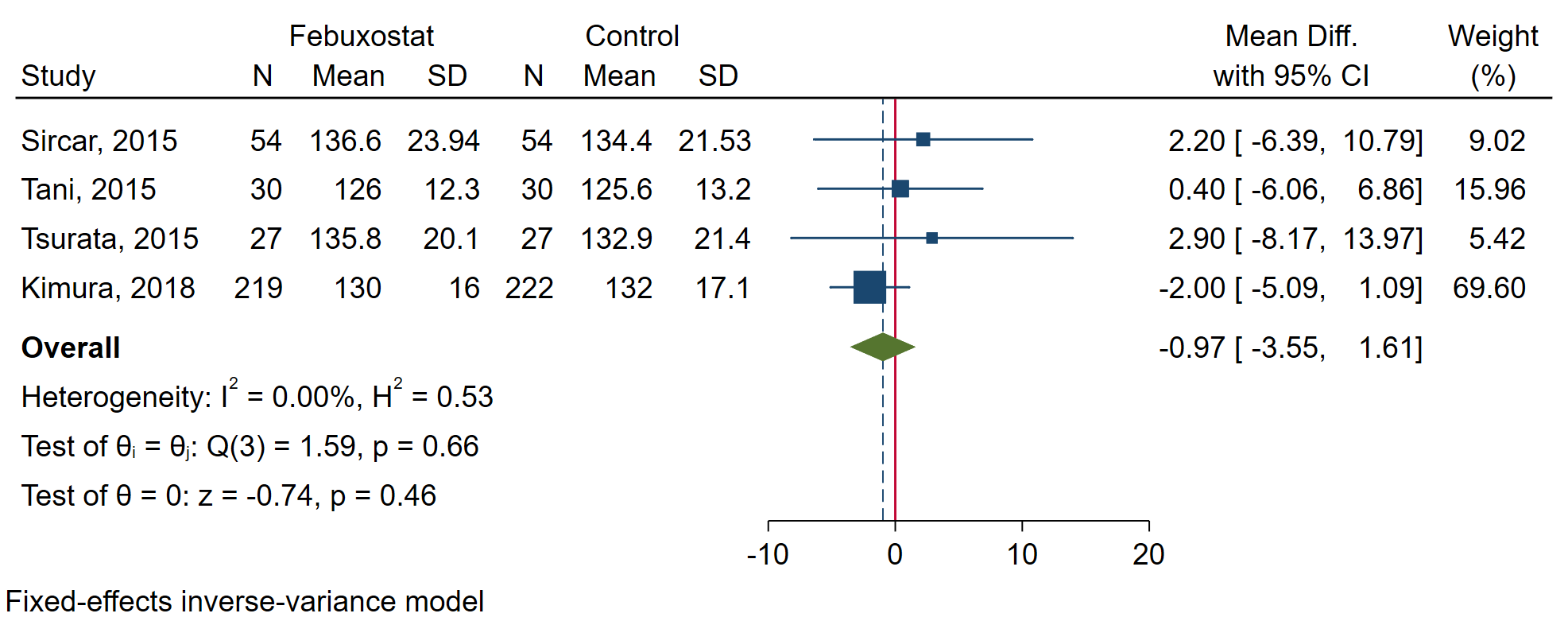


c) febuxostat vs allopurinol


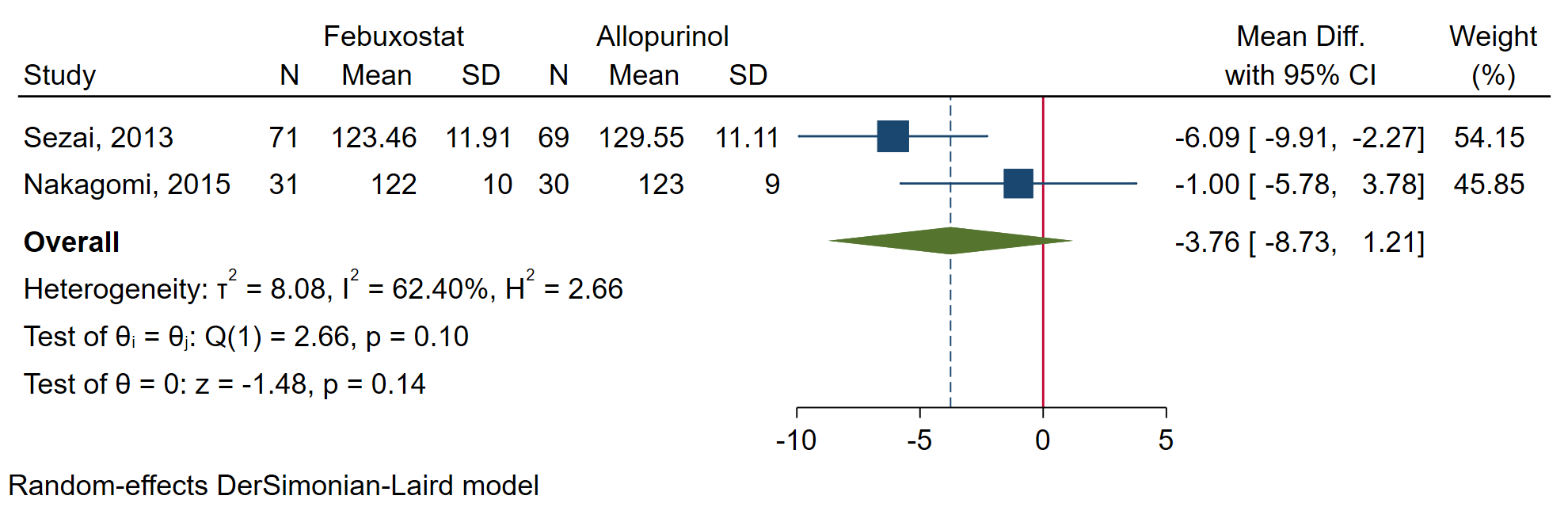


CI, confidence interval; Mean Diff., mean difference; SD, standard deviation; ULT, urate-lowering therapy.

**Figure S6.** Forest plots of adverse events

a) allopurinol vs placebo/no ULT


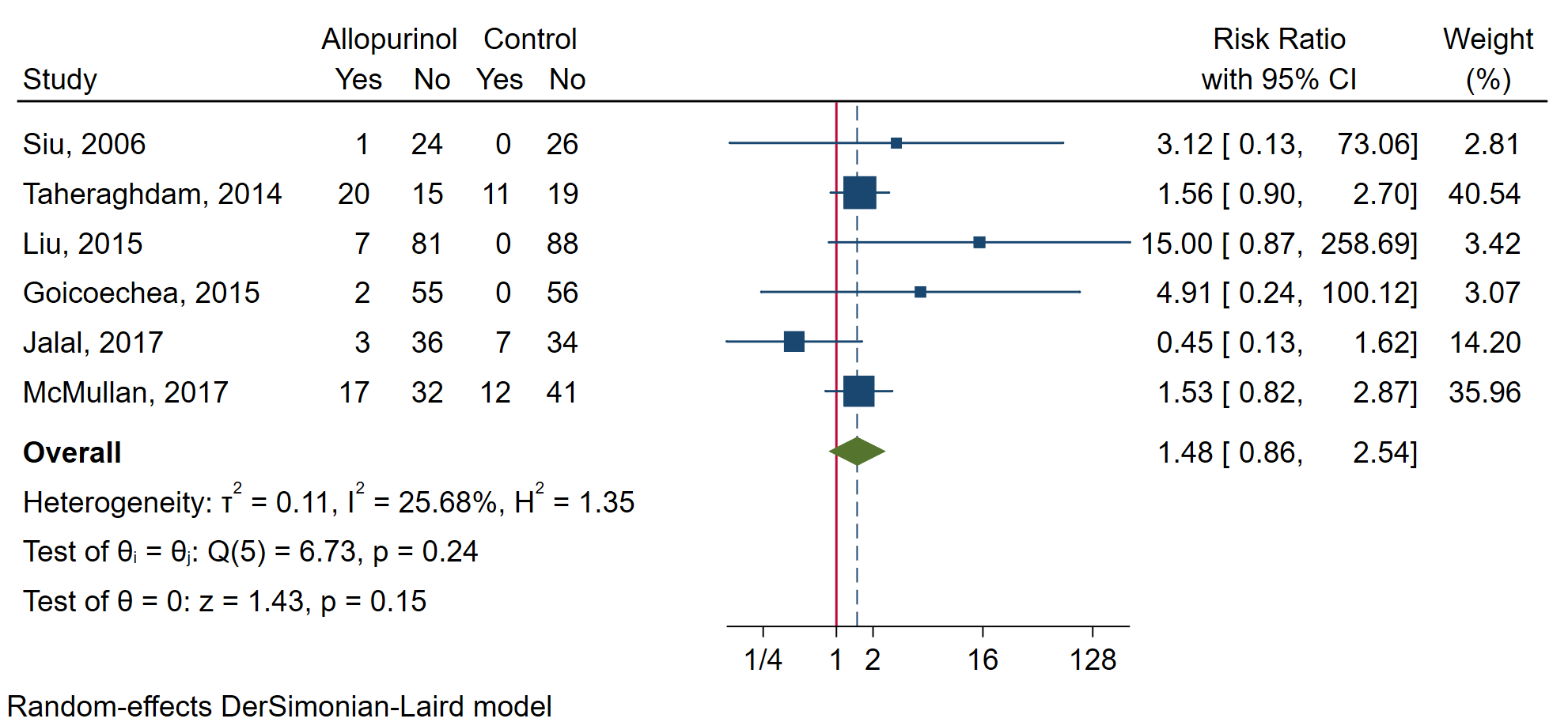


b) febuxostat vs placebo/no ULT


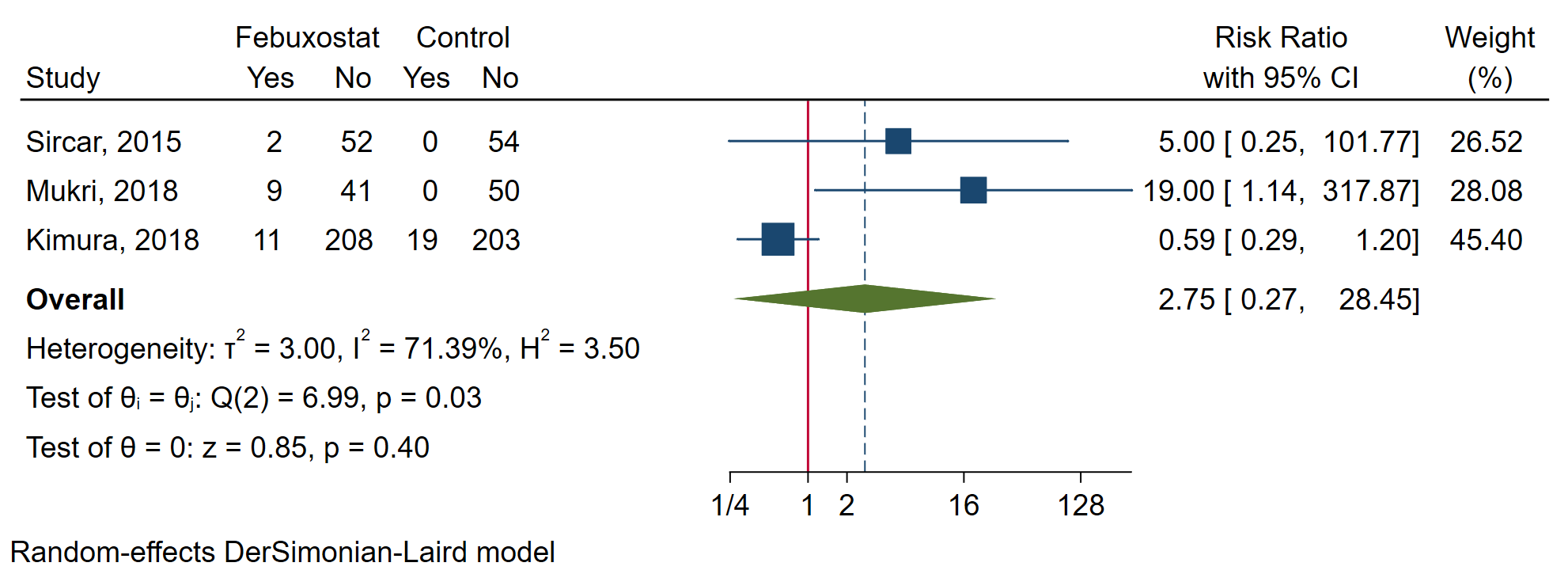


c) febuxostat vs allopurinol


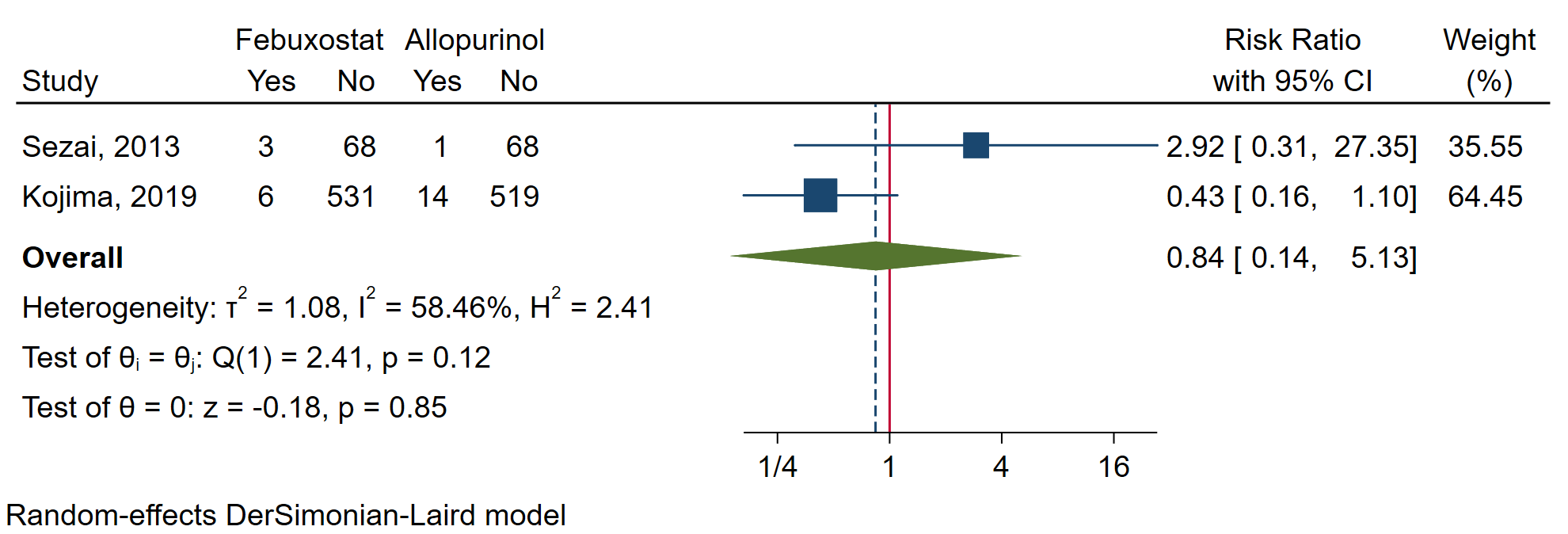


CI, confidence interval; ULT, urate-lowering therapy.

**Figure S7.** Forest plots: subgroup analysis of serum urate level by proportion of patients with hypertension


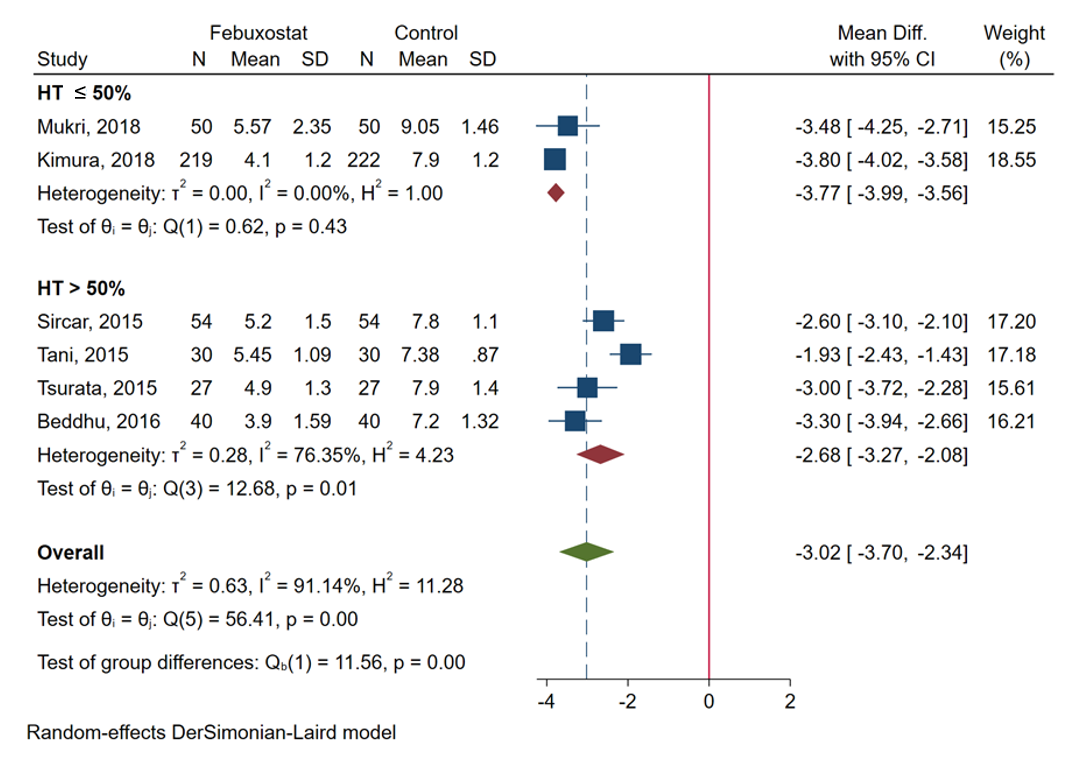


CI, confidence interval; HT, hypertension; Mean Diff., mean difference; SD, standard deviation

**Figure S8.** Forest plots: sensitivity analysis of serum urate level excluding study containing all hypertensive patients.


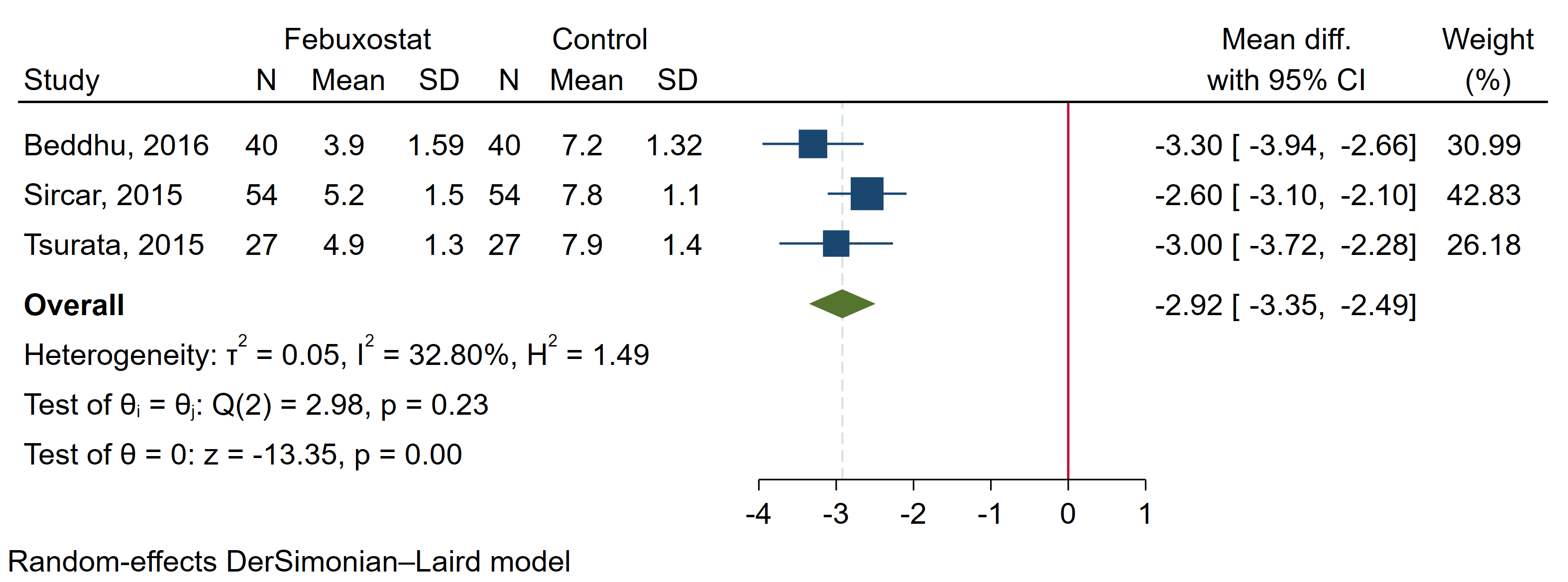


CI, confidence interval; HT, hypertension; Mean Diff., mean difference; SD, standard

**Figure S9.** Network maps of primary outcomes


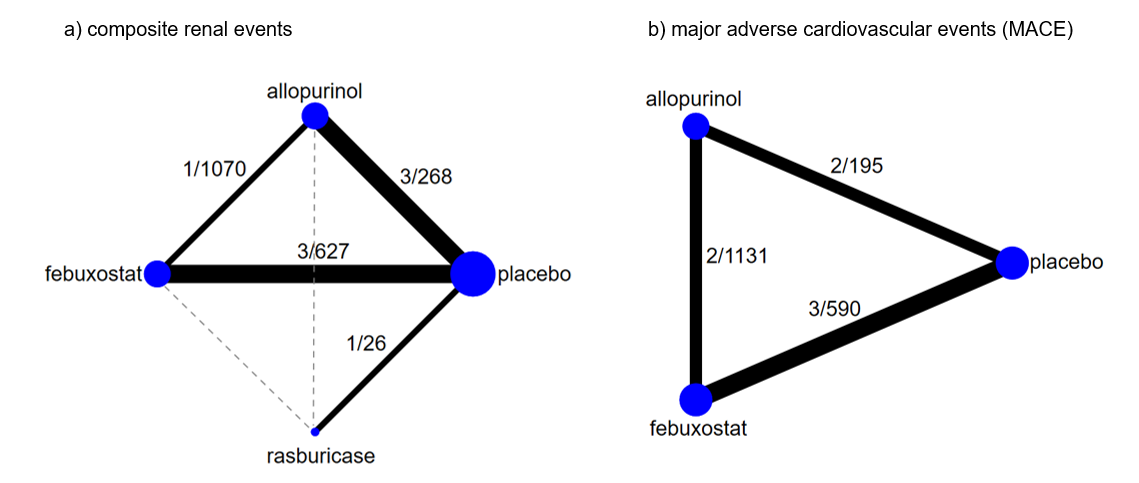


Network maps of composite renal events and MACE. Size of nodes was weighted by number of studies. Size of edges was weighted by number of subjects in each comparison. Each number on plots represents numbers of comparison arms and subjects contributing to the corresponding comparison. Dashed edges represent indirect comparison by network.

**Figure S10.** Network maps of secondary outcomes


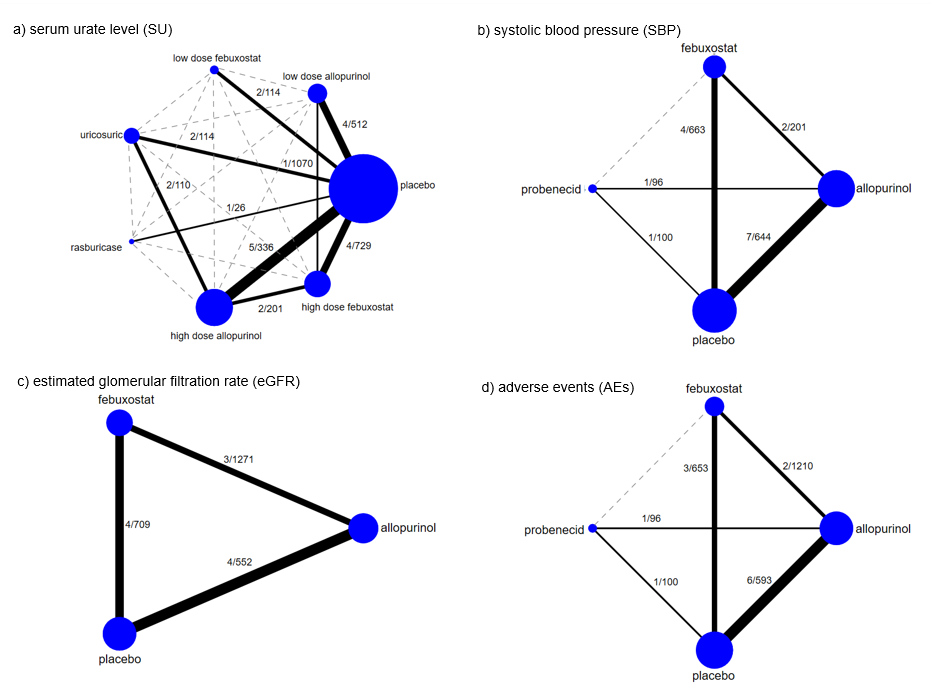


Network maps of SU, SBP, eGFR, and AEs. Size of nodes was weighted by numbers of studies. Size of edges was weighted by number of subjects in each comparison. Each number on plots represents numbers of comparison arms and subjects contributing to the corresponding comparison. Dashed edges represent indirect comparison by network.

**Figure S11.** Comparison-adjusted funnel plots


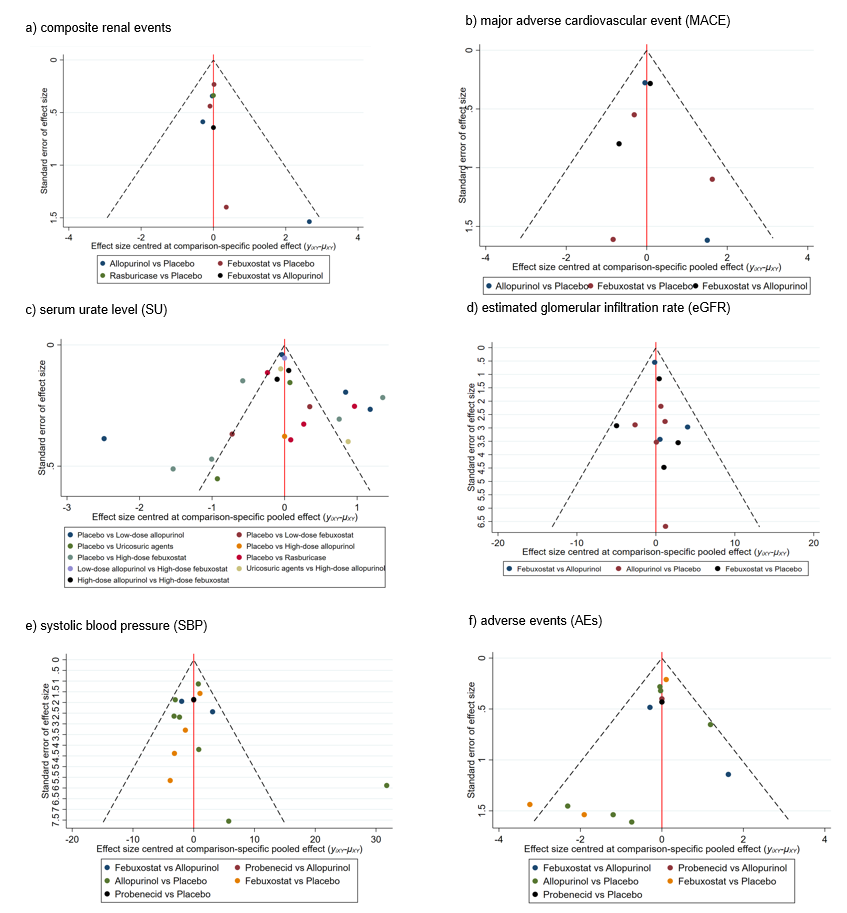


**Figure S12.** Scatter plot of the surface under the cumulative ranking curve for highest efficacy and lowest adverse events of urate-lowering agents


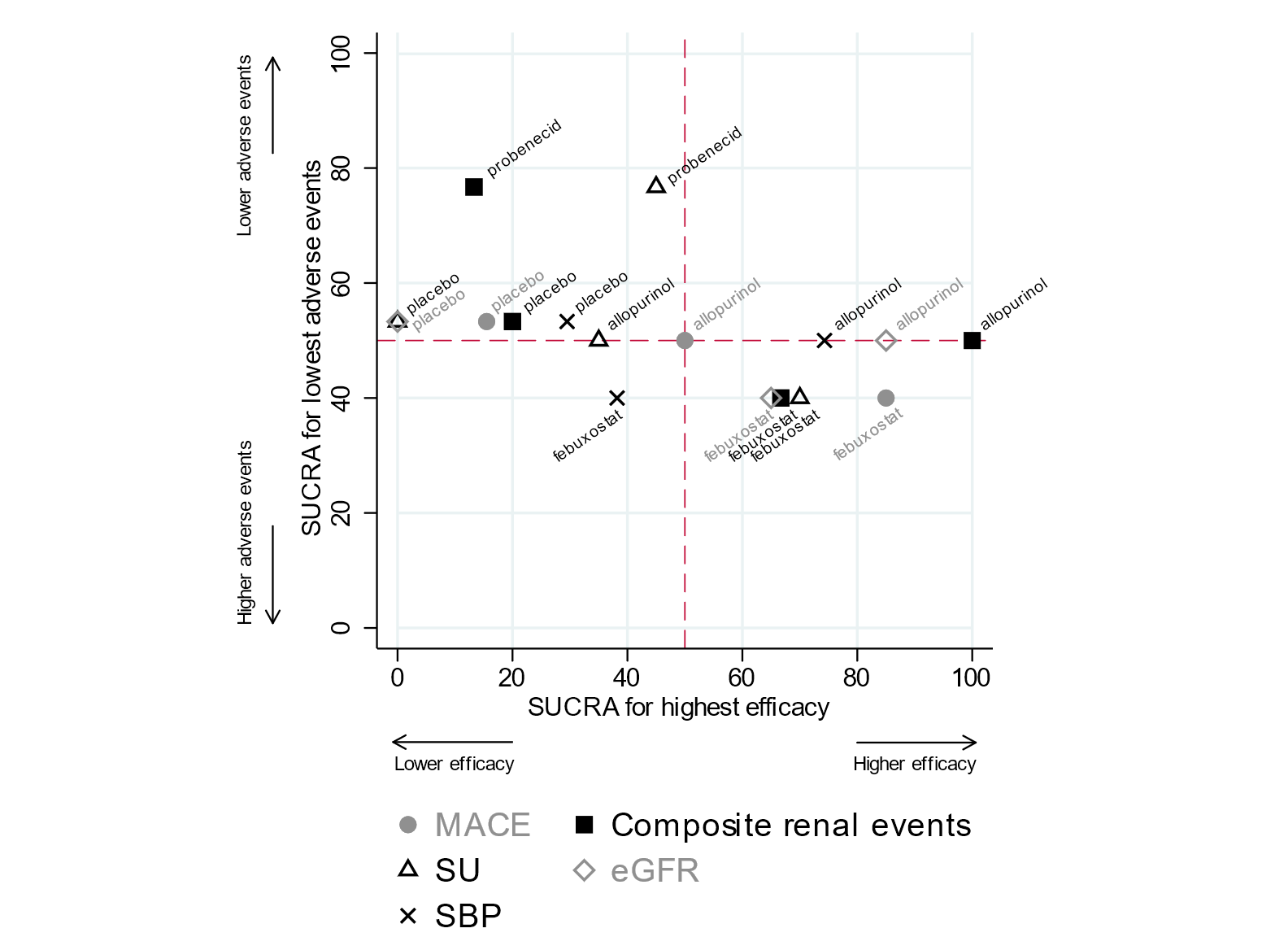


eGFR, estimated glomerular filtration rate; MACE, major adverse cardiovascular events; SBP, systolic blood pressure; SU, serum urate level; SUCRA, surface under the cumulative ranking curve.

**Figure S13.** Network forest plot


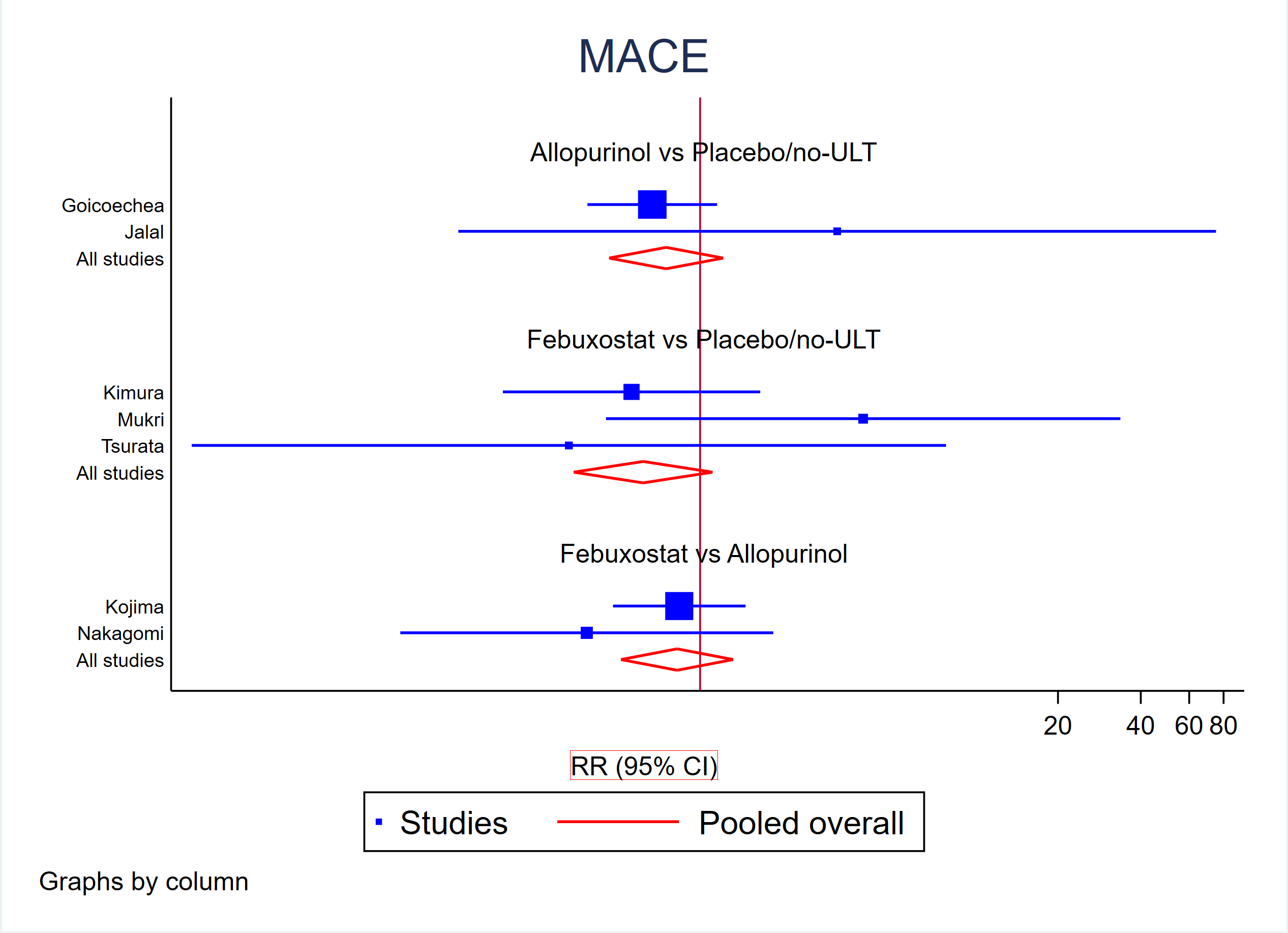

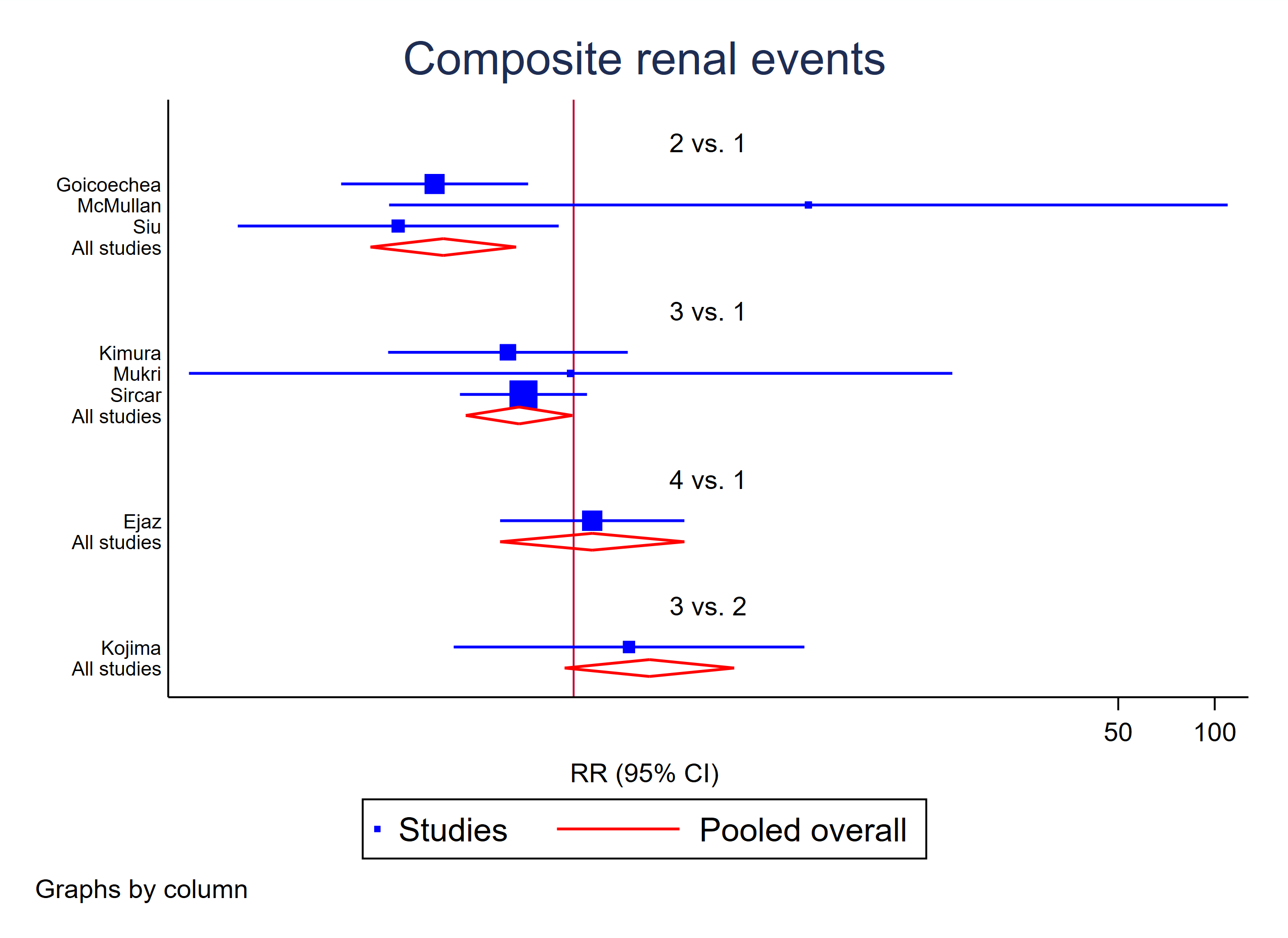


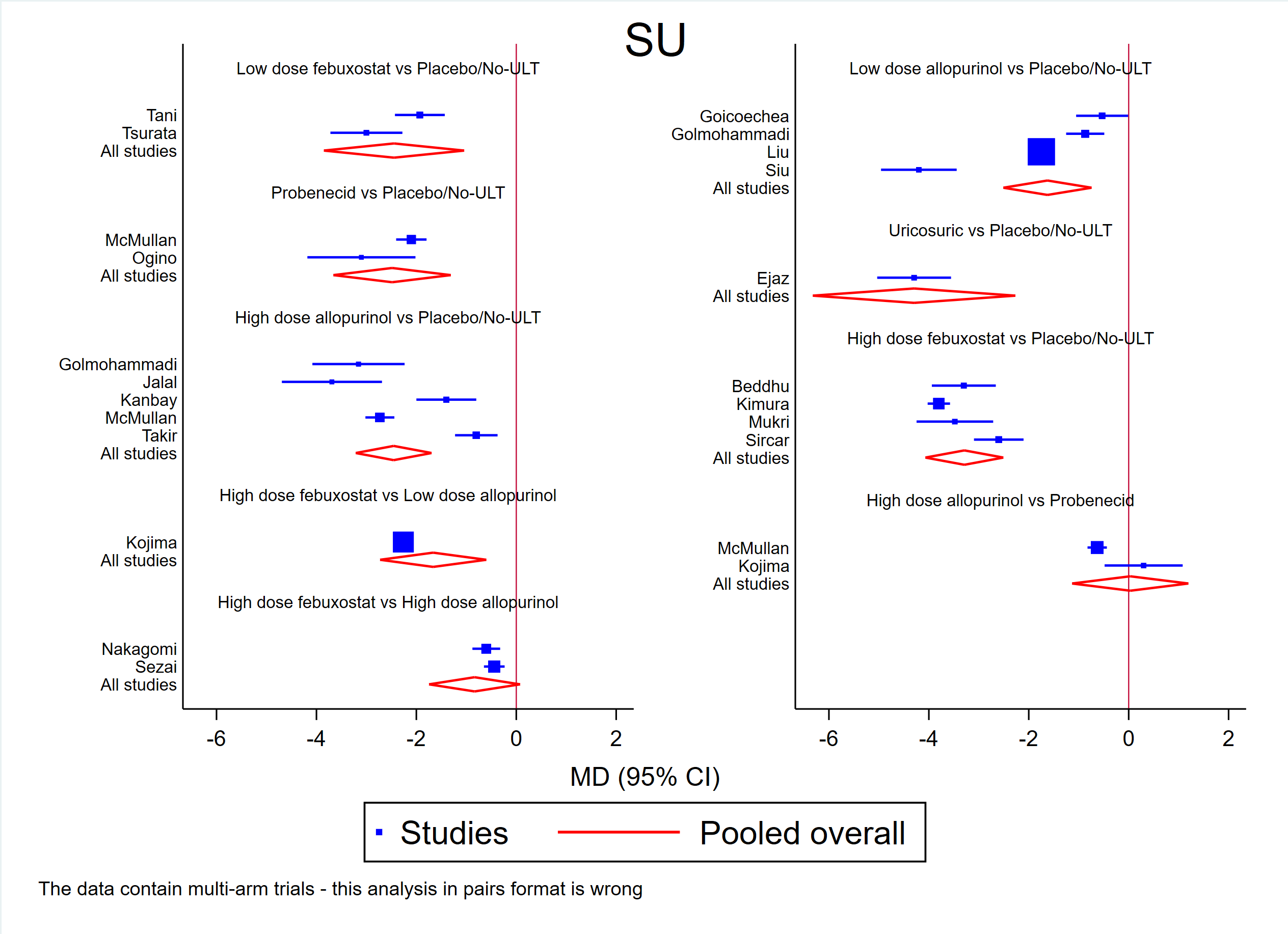

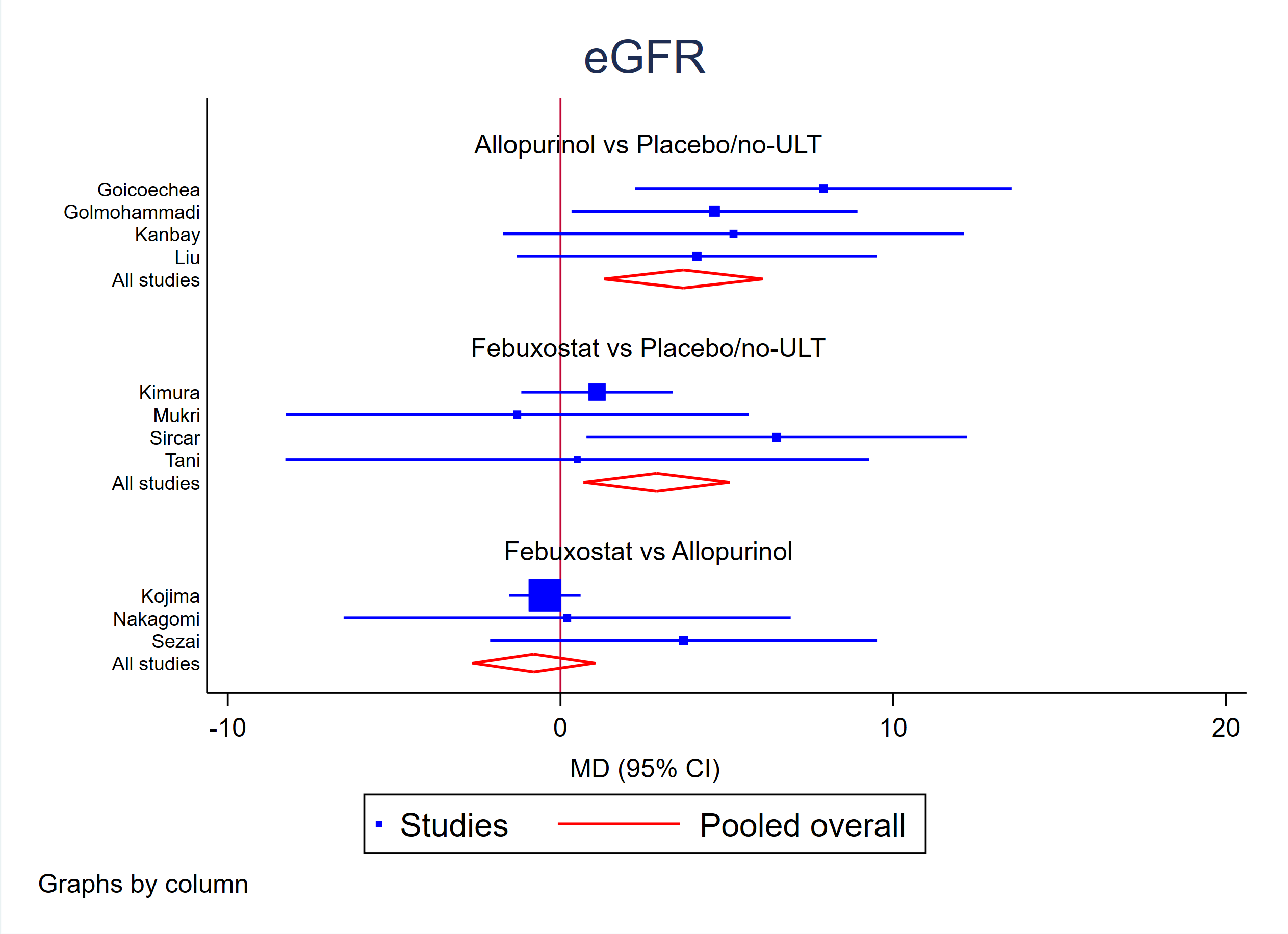


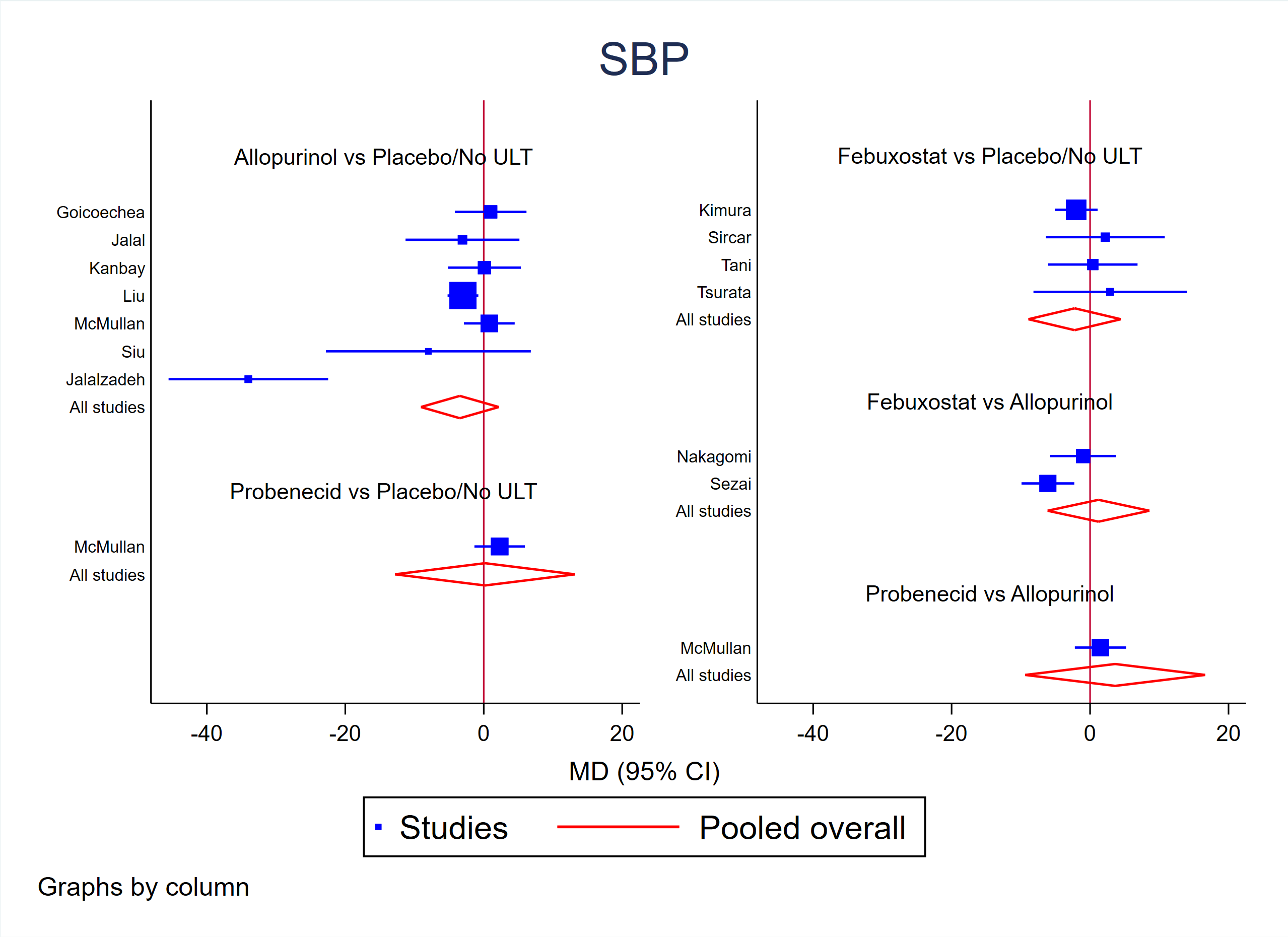

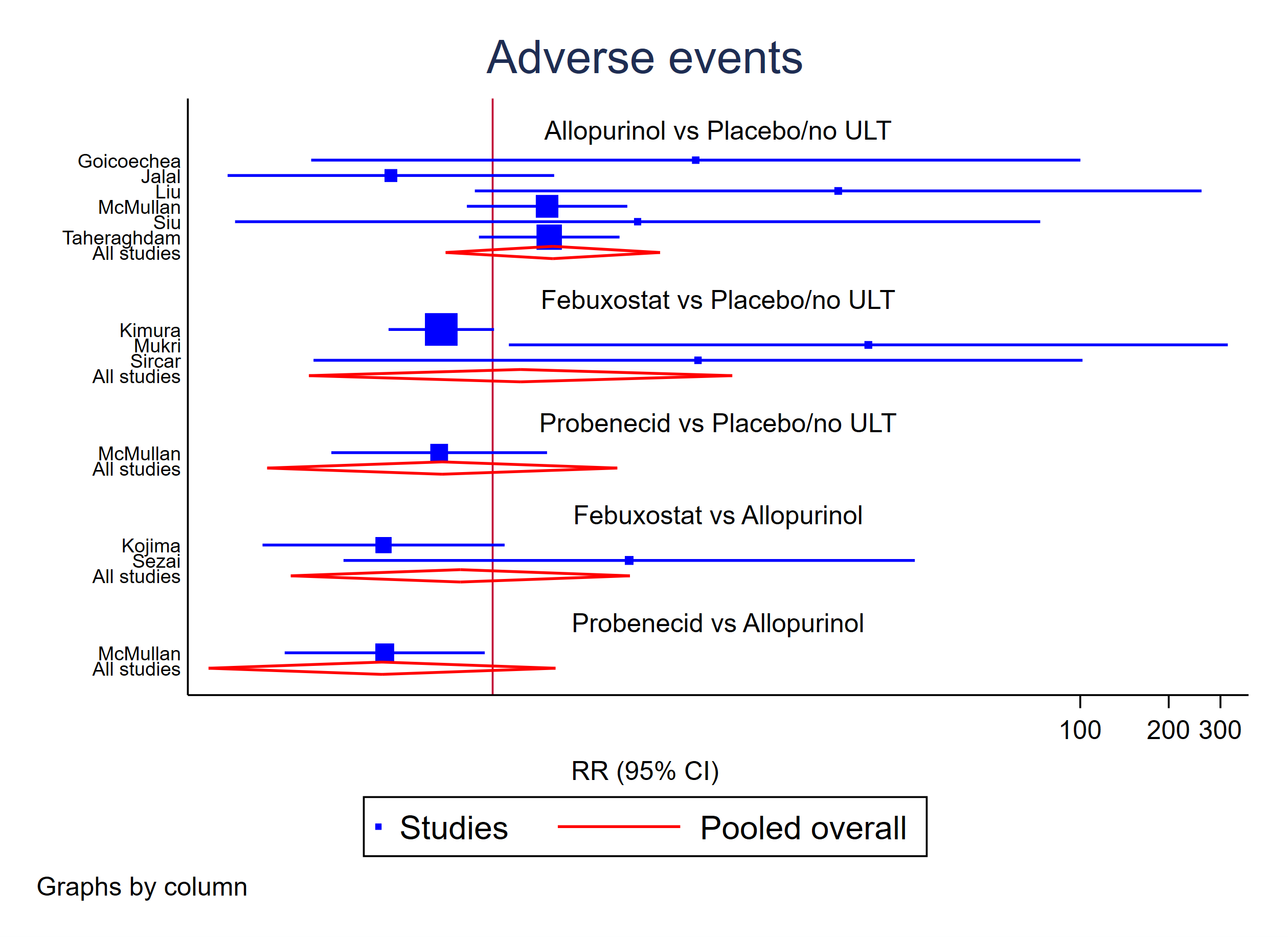

Supplement: Supplementary file 1 — Additional file 1. [file 12882_2022_2850_MOESM1_ESM.docx]
